# Bat-associated *Trypanosoma* diversity, geographic extension of known clades, and predominance of *T. cruzi* TcIV in a conserved tropical forest of southeastern Mexico

**DOI:** 10.1007/s00436-026-08670-w

**Published:** 2026-04-10

**Authors:** Martha P. Ibarra-López, Román Espinal-Palomino, César R. Rodríguez-Luna, Joel Moo-Millán, Etienne Waleckx, Rodolfo Dirzo, Salvador Montiel, Víctor M. Vidal-Martínez, Carlos N. Ibarra-Cerdeña

**Affiliations:** 1 Department of Human Ecology, Center for Research and Advanced Studies of the National Polytechnic Institute, Mérida, Mexico; 2 Centro de Investigaciones Regionales “Dr. Hideyo Noguchi”, Universidad Autónoma de Yucatán, Mérida, México; 3 Institut de Recherche pour le Développement, UMR INTERTRYP IRD, CIRAD, Université de Montpellier, Montpellier, France; 4 Departments of Biology and Earth Systems Science, Stanford University, Stanford, CA USA; 5 Deparment of Sea Resources, Center for Research and Advanced Studies of the National Polytechnic Institute, Mérida, Mexico; 6 IMIPAS, Secretaría de Agricultura y Desarrollo Rural, Ciudad de México, México

**Keywords:** Bats, Sylvatic transmission, *Triatoma dimidiata*, Tropical dry forest, *Trypanosoma cruzi*, Rodent reservoirs

## Abstract

Trypanosomatids of the *Schizotrypanum* clade display high ecological and evolutionary diversity, yet their sylvatic cycles remain understudied in many Neotropical biomes. We conducted the first integrative molecular survey of *Trypanosoma* diversity in a conserved seasonal tropical forest in the Yucatán Peninsula, Mexico. We screened *Triatoma dimidiata* vectors and small mammal hosts (rodents and bats) using satellite DNA (SatDNA) for *Trypanosoma cruzi* detection, Miniexon PCR for *T. cruzi* discrete typing unit (DTU) identification, and 18 S rRNA sequencing to identify other *Trypanosoma* species in bats. *Trypanosoma cruzi* was detected by SatDNA in 81.8% of collected vectors (95% CI: 75.07–87.38%; *n* = 165) and in 46.6% of rodents (95% CI: 36.7–56.7%, *n* = 103) and in 46.2% of bats (95% CI: 38.56–53.97%; *n* = 171). Discrete typing unit (DTU) analysis revealed that *T. cruzi* DTU IV (TcIV) predominated in the conserved tropical forest, being mainly associated with vectors and rodents, whereas TcI was more frequent in bats. Phylogenetic reconstruction based on 18 S rRNA revealed multiple bat-associated lineages, including sequences related to *T. wauwau*, *T. madeirae*, and Neobat clades—reported here for the first time in Mexico. Our results underscore the role of protected forests as reservoirs of trypanosomatid diversity and the relevance of multi-host surveillance to better understand transmission dynamics. This study contributes novel insights into the ecology, biogeography, and evolutionary history of *Trypanosoma* spp. in Mesoamerica and supports efforts to strengthen early detection of potential zoonotic threats.

**Graphical Abstract:**

**Figure:**
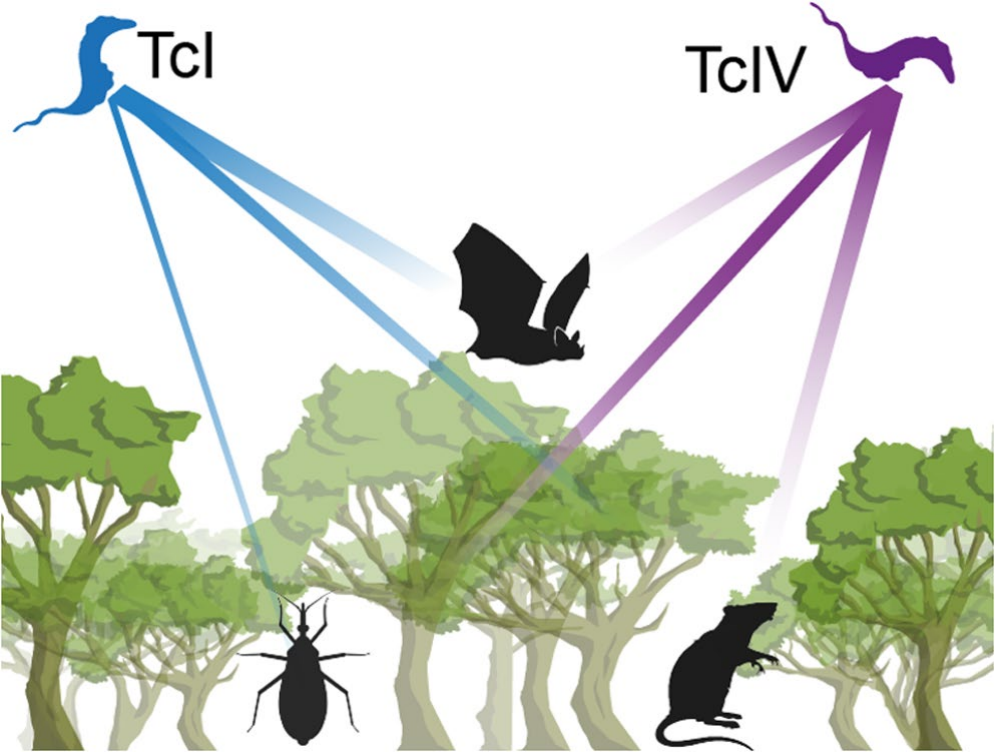
Schematic representation of *Trypanosoma cruzi* discrete typing unit (DTU) associations in a sylvatic transmission cycle within a conserved seasonal tropical forest in Yucatán, Mexico. TcI (blue) was predominantly associated with bats (highly mobile hosts), while TcIV (purple) was primarily detected in rodents and triatomine vectors (low-dispersal hosts), illustrating distinct host-pathogen interaction patterns that reflect ecological filtering of *T. cruzi* lineages.

**Supplementary Information:**

The online version contains supplementary material available at 10.1007/s00436-026-08670-w.

## Introduction

Trypanosomatids (family Trypanosomatidae) are a diverse group of kinetoplastid protozoa, many of which are responsible for significant diseases in humans, domestic animals, and wildlife (Kaufer et al. 2017). These unicellular flagellates are characterized by the presence of a kinetoplast, a unique mitochondrial DNA structure, and a complex life cycle that often involves both invertebrate and vertebrate hosts (Hoare 1972; Vickerman 1976). Within this family, the genera *Trypanosoma* and *Leishmania* include species of major medical and veterinary importance, such as *Trypanosoma brucei*, the causative agent of African trypanosomiasis, *T. cruzi*, responsible for Chagas disease, and various *Leishmania* species that cause leishmaniases (Lawyer and Perkins 2000; Stuart et al. 2008). Among vertebrates, mammals represent the most significant reservoirs, particularly in the case of *Trypanosoma* and *Leishmania*, but reptilian and amphibian hosts also harbor diverse trypanosomatid species, indicating deep evolutionary associations with a broad range of ectothermic and endothermic hosts (Guhl and Vallejo 2003; Ferreira et al. 2007). The host spectrum and transmission strategies of trypanosomatids are thought to have played a key role in their diversification, with vector-host interactions shaping their evolutionary trajectories and driving adaptations such as antigenic variation to evade host immune responses (Stevens et al. 1999; Stevens and Gibson 1999).

The Neotropical region harbors remarkable diversity of bat-associated *Trypanosoma* species, reflecting the ecological and evolutionary complexity of host–parasite interactions in this biodiverse landscape. In recent years, several bat trypanosomes have been described, including *Trypanosoma wauwau*, *T. madeirae*, and members of the Neobat clade, identified across diverse bat families such as Phyllostomidae and Vespertilionidae (Cottontail et al. 2009; Lima et al. 2015b; Barros et al. 2019). These lineages have been reported from multiple countries in South and Central America, including Brazil, Colombia, French Guiana, and Mexico (Ramírez et al. 2014; Alves et al. 2021), suggesting a broad geographic distribution within Neotropical bat communities.

Advances in molecular approaches, particularly 18 S ribosomal RNA (rRNA) gene sequencing, have substantially improved the detection and phylogenetic placement of bat trypanosomes. The 18 S rRNA marker, which combines conserved and variable regions, has proven especially useful for resolving evolutionary relationships among *Trypanosoma* species while maintaining taxonomic comparability across studies (Hughes and Piontkivska 2003; Lima et al. 2015b). Continued molecular surveys are therefore essential to refine our understanding of the diversity, host associations, and biogeographic patterns of Neotropical bat trypanosomatids.

Bats are widely recognized as important reservoirs of diverse *Trypanosoma* species, and phylogenetic analyses indicate that several bat-associated lineages are closely related to *T. cruzi*, (Hamilton et al. 2012; Lima et al. 2015a). Moreover, evidence of vertical transmission of *T. cruzi* in wild bats (Añez et al. 2009) supports the hypothesis of a long-standing evolutionary association between this parasite and chiropteran hosts.

The transition from a bat-restricted ancestor to a parasite capable of infecting a wide range of mammalian hosts may have been facilitated by changes in vector ecology, as triatomine bugs—primary vectors of *T. cruzi*—are generalist feeders capable of feeding on multiple host species in sylvatic environments (Jansen et al. 2018). This host-switching scenario is supported by phylogenomic studies showing that *T. cruzi* exhibits substantial genetic diversity, structured into six multiple discrete typing units (DTUs) that reflect complex evolutionary histories, ecological adaptations, and host associations (Zingales and Macedo 2023). In addition, recent evidence shows that vertical transmission can sustain infection and contribute to parasite diversity in mammalian reservoirs even in habitats where vectors are rare or absent (Gibson et al. 2025). The evolutionary history of *T. cruzi* thus highlights the role of bats as key ancestral hosts, with vector-mediated host-switching events facilitating the emergence of a highly adaptable and widely distributed parasite.

Accumulating evidence from Mexico and other Neotropical regions demonstrates that the dispersion of *T. cruzi* in sylvatic environments is geographically widespread yet ecologically structured, with distinct DTUs exhibiting differential distributions across biomes, vector species, and mammalian host assemblages. For example, TcI predominates across Mesoamerica, where it is frequently associated with arboreal and generalist mammals—including bats—and vectors such as *T. dimidiata*, whereas TcII, TcIV, and TcVI occur within more heterogeneous transmission networks involving terrestrial reservoirs and mixed infections in sylvatic triatomines (Izeta-Alberdi et al. 2016; Díaz-Valdez et al. 2021; Zingales and Macedo 2023). Although TcI remains the most widespread DTU in the Americas and is the dominant lineage in North America, the remaining DTUs (Tc II-VI) account for less than 20% of records in this region; notably, in Central America, DTU distribution is overwhelmingly dominated by TcI (93%) and TcIV (7%) (Brenière et al. 2016).

Given the ecological importance of bats, rodents, and triatomine insects as hosts and vectors of trypanosomatids, this study investigates the diversity and phylogenetic relationships of *Trypanosoma* species circulating in a conserved tropical dry forest of the Yucatan Peninsula. Although the Mesoamerican region is recognized as a biodiversity hotspot (Myers et al. 2000), the diversity and transmission dynamics of trypanosomatids infecting local mammalian hosts and insect vectors remain poorly understood. Recent studies from the peninsula have reported a broader diversity of *Trypanosoma* species, particularly from bats and other mammals in human-modified landscapes (Moo-Millán et al. 2024). However, surveys in mature forest habitats—where host diversity is typically higher—remain scarce, and thus it is plausible that trypanosomatid diversity has been underestimated. By integrating molecular detection with phylogenetic analyses, this study aims to characterize the diversity of *Trypanosoma* species in a conserved tropical forest. Using 18 S rRNA and additional molecular markers, we investigate the host range, transmission cycles, and evolutionary relationships of trypanosomatids. Furthermore, by identifying *T. cruzi* discrete typing units (DTUs) in small mammals (rodents and bats) and triatomine vectors, we aim to elucidate potential host–parasite networks operating within a conserved tropical forest landscape. This forest is situated in a region surrounded by Mayan communities where Chagas disease remains endemic and has been historically under-recognized as a rural public health issue (Aké-Chan et al. 2025). In this area, *T. dimidiata* (*sensu lato*) is the principal vector (Montes de Oca-Aguilar et al. 2022), ranking among the most widespread and epidemiologically significant vectors of *T. cruzi* in Mexico (Ramsey et al. 2015) and exhibiting high abundance both within forested areas and rural villages (Moo-Millán et al. 2023).

Our focus on small mammals as *Trypanosoma* hosts is driven by two main considerations. First, bats are recognized as evolutionary reservoirs of trypanosomatid diversity and play a key role in maintaining members of the *T. cruzi* clade (Austen and Barbosa 2021). Second, rodents are among the most important reservoirs of *T. cruzi* in Mesoamerica (Ibarra-Cerdeña et al. 2024). The ecological traits of both groups (i.e. bats’ high mobility and rodents’ resilience to habitat disturbance), make them particularly relevant as potential amplifiers of *T. cruzi* transmission across forest–village interfaces.

Our findings offer novel insights into the sylvatic ecology of trypanosomatids in conserved tropical dry forests, emphasizing the role of wildlife in maintaining transmission cycles and informing future strategies for zoonotic disease surveillance and prevention.

## Materials and methods

### Ethical statements

All wild mammal captures and handling procedures were conducted in compliance with ethical standards and under approved collection permits (Secretaría de Medio Ambiente y Recursos Naturales, SEMARNAT). To ensure the welfare of the animals, we followed standard ethical protocols for field sampling, including the use of species-appropriate live traps (Sherman and mist nets), regular monitoring to minimize stress, and safe handling by trained personnel using personal protective equipment (Sikes et al. 2016). Pregnant females, both rodents and bats, were released immediately after capture and were not subjected to invasive procedures. Euthanasia of rodents and bats was performed using humane methods in accordance with the guidelines of the American Society of Mammalogists (Sikes et al. 2016), ensuring rapid and painless procedures. All efforts were made to minimize suffering, and only the minimum number of animals necessary for the study was collected.

### Study site

**Fig. 1:**
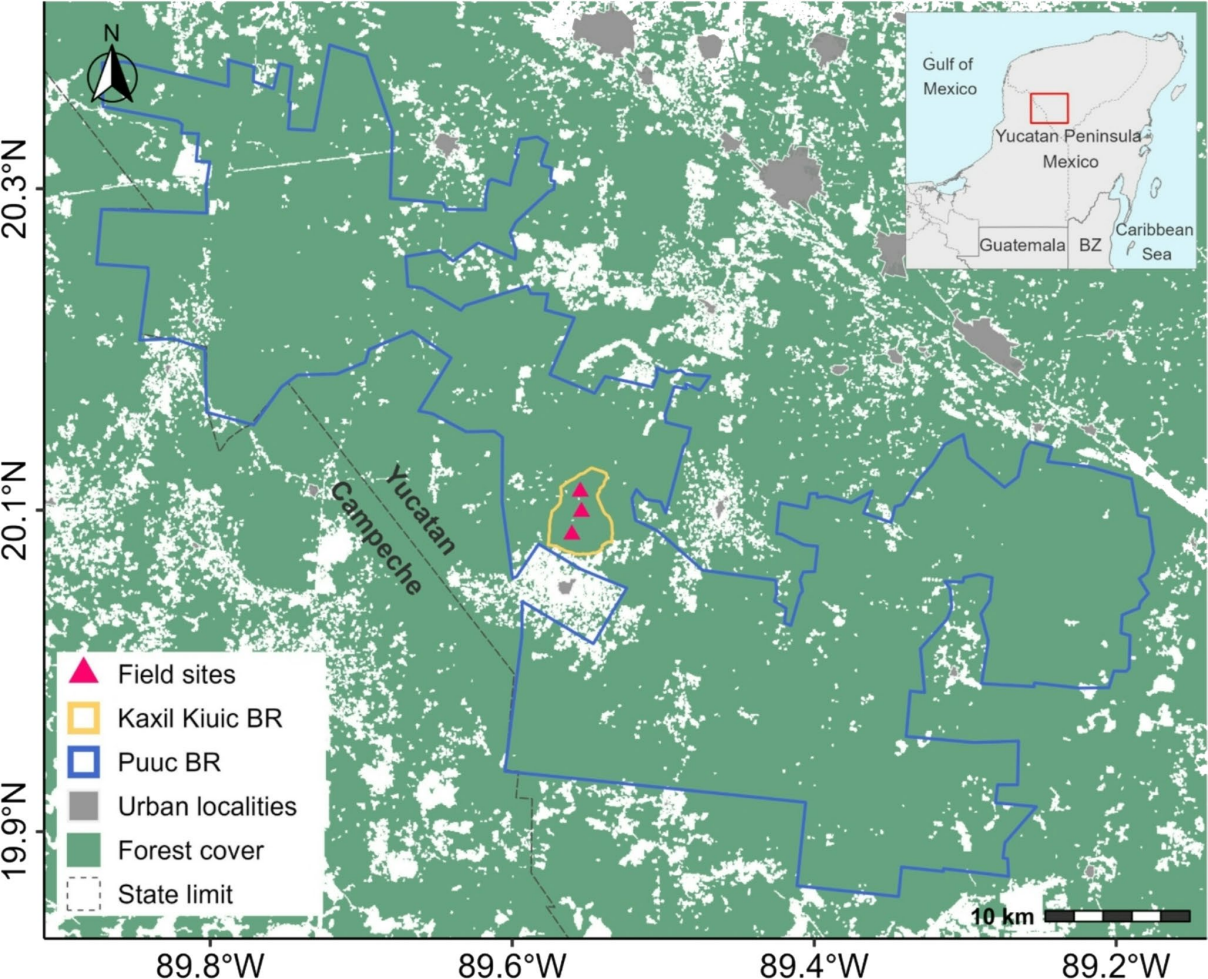
Map of the study area in the tropical dry forest region of the Yucatán Peninsula, southeastern Mexico. Triangles indicate field sampling sites where vectors and mammalian hosts were captured. The study was conducted within and around the Kaxil Kiuic Biocultural Reserve (yellow outline), situated inside the larger Puuc Biocultural Reserve (blue outline). Forest cover is shown in green, while urban localities are represented in gray. The state boundary between Yucatán and Campeche is marked with a dashed line. The inset map provides the regional context of the study site within Mexico and northern Central America

The study was conducted in a well-preserved forest within the Kaxil Kiuic Biocultural Reserve (KKBR), located in the southern region of Yucatán State, Mexico. The reserve encompasses approximately 1,600 hectares of low to medium semi-deciduous forest, with an average canopy height of 25–30 m, comprising a mosaic of mature (> 100 years) and younger (20–40 years) forest stands (George-Chacón et al. 2022). The KKBR is part of the El Puuc State Biocultural Reserve network, a regional conservation corridor that includes other key areas of biocultural importance across the Yucatán Peninsula. The reserve plays a critical role in preserving both biodiversity and cultural heritage, supporting forest heterogeneity and landscape-scale ecological connectivity. A detailed description of the study area is available in Montes de Oca-Aguilar et al. (2024) and León et al. (2025). Sampling of bats, rodents, and vectors was carried out at three sites within the KKBR, each separated by approximately 1 km (Fig. 1).

### Sampling design and effort

Fieldwork was conducted between 2014 and 2019 in a conserved tropical dry forest in southeastern Mexico. Sampling targeted three biological groups, triatomine vectors, rodents, and bats, to assess the diversity and host associations of *Trypanosoma* spp. Sampling effort varied across years for each taxon due to logistical constraints and climatic variability inherent to long-term fieldwork in tropical environments (Table S1). As a result, comparisons among host groups and years were based on the number of infected individuals rather than total captures and were interpreted with caution. All sampling campaigns were conducted during the dry season, when vector activity is typically highest (Moo-Millán et al. 2023).

Triatomine bugs were collected using modified light traps deployed at night. Each trap consisted of a 3 × 3 m white cloth illuminated by two 1000-lumen LED light sources and operated for four hours per night. Trapping effort varied annually between 2014 and 2019 and ranged from 1 to 14 sampling nights per year, with a constant effort of 4 trap-hours per night. A total of 31 trap-nights were conducted during the study period, resulting in 124 trap-hours of sampling effort. The lowest effort occurred in 2014 and 2018 (1 night each), while the highest effort was recorded in 2019 (14 nights). All captured triatomines were morphologically identified, sexed when adults, and classified by developmental stage (nymph or adult). Specimens were identified to species level using standard morphological dichotomous keys for Triatominae (Lent and Wygodzinsky 1979), supported by the electronic identification tool TriatoDex (Gurgel-Goncalves et al. 2021) and Cruz et al. (2021). Bugs were individually preserved in 96% ethanol, and ethanol was injected into the abdominal cavity to improve preservation of the digestive tract for molecular analyses.

Rodents were captured using Sherman live traps deployed between 2015 and 2019. Trapping effort varies annually from 60 to 90 traps per year, operated for 4 to 7 consecutive nights, depending on the sampling period. Captured rodents were weighed, sexed, and assessed for reproductive condition. Morphometric measurements were recorded to support species identification. Taxonomic identification followed Álvarez-Castañeda et al. (2017) and Reid (2009). From each individual, either a blood sample (30 µL) or heart tissue was collected. Heart tissues were preserved in 96% ethanol and stored frozen until molecular analysis.

Bats were sampled between 2015 and 2019 using mist nets placed in the forest understory. Sampling effort varied substantially among years and was quantified as net length (meters) per night. Annual effort ranged from 126 to 648 mist-net meters per night, deployed over 2 to 7 nights per year. Each captured bat was measured, weighed, sexed, and assessed for reproductive condition. Taxonomic identification followed Medellín et al. (2008) and Reid (2009). Blood samples (30 µL) or heart tissue were collected for molecular analyses. Heart tissues were preserved in 96% ethanol and stored at freezing temperatures. Pregnant females were released immediately after capture.

### Nucleic acid extraction

Genomic DNA was extracted using the DNeasy Blood & Tissue Kit (Qiagen, Valencia, CA, USA), following the manufacturer’s instructions. Extractions were performed from approximately 25 mg of cardiac tissue collected from bats and rodents, as well as from the intestinal contents of *T. dimidiata*. The final DNA elution was carried out in 100 µL of DNase/RNase-free water and stored at − 20 °C until further analysis. To confirm DNA extraction, DNA quantification was performed using a Nanodrop™ 1000 spectrophotometer (Thermo Scientific, Waltham, MA, USA).

### Detection of *Trypanosoma* spp.

PCR amplifications were performed in a final reaction volume of 25 µL, consisting of 12.5 µL of GoTaq^®^ Green Master Mix (Promega, Madison, WI, USA), 1 µL of each primer (10 µM), 7.5 µL of ultrapure water, and 3 µL of genomic DNA. As internal controls, TcVI (CL-Brener) was used as a positive control, and 3 µL of nuclease-free water from the GoTaq^®^ Green Master Mix kit (Promega, Madison, WI, USA) was used as a negative control in place of the DNA template. Positive and negative internal amplification controls were included in each PCR run to ensure reaction integrity and to rule out contamination or inhibition. All reactions were conducted in a Veriti™ 96-well thermal cycler (Applied Biosystems™, Foster City, CA, USA).

### Molecular detection and characterization of *Trypanosoma* spp.

To screen for *T. cruzi* infection, the nuclear satellite DNA (SatDNA) marker was amplified using the primers Cruzi 1 (5′-AST CGG CTG ATC GTT TTC GA-3′) and Cruzi 2 (5′-AAT TCC TCC AAG CAG CGG ATA-3′), targeting a ~ 195 bp fragment as described by Schijman et al. (2011). PCR conditions consisted of an initial denaturation at 95 °C for 15 min; followed by 35 cycles of denaturation at 94 °C for 10 s, annealing at 60 °C for 30 s, and extension at 72 °C for 30 s; with a final extension at 72 °C for 10 min.

To identify *T. cruzi* discrete typing units (DTUs), DNA amplifications were performed following the protocol of Aliaga et al. (2011), which validated the use of a miniexon-based multiplex PCR for rapid DTU discrimination. The method utilizes three primers targeting the intergenic region of the *T. cruzi* miniexon gene (Tc1: 5′-ACACTTTCTGTGGCGCTGATCG-3′, Tc2: 5′-TTGCTCGCACACTCGGCTGCAT-3′, and Tc3: 5′-CGCGWACAACCCCTMATAAAAATG-3′), one primer specific to *T. rangeli* (Tr: 5′-CCTATTGTGATCCCCATCTTCG-3′), and a common downstream primer (Me: 5′-TACCAATATAGTACAGAAACTG-3′) located in a conserved region of the miniexon gene (Fernandes et al. 2001). The PCR conditions consisted of an initial denaturation at 94 °C for 3 min, followed by 35 cycles of denaturation at 94 °C for 1 min, annealing at 56 °C for 1 min, and extension at 72 °C for 1 min, with a final extension at 72 °C for 5 min. Amplified products were resolved on 3% agarose gels.

DTUs were identified by characteristic product sizes: TcI (~ 200 bp), TcII/TcV/TcVI (~ 250 bp), and TcIII/TcIV (~ 150 bp), as described by Aliaga et al. (2011). Because TcI can be reliably identified by its ~ 200 bp band, PCR products showing other fragment sizes or ambiguous patterns were subjected to Sanger sequencing to confirm DTU identity. Forward and reverse sequences were assembled and aligned using the MAFFT algorithm implemented in Geneious Prime v2025.0.2. Alignments were manually inspected, and any discrepancies were resolved by examination of the corresponding chromatograms. Taxonomic identity was inferred using BLAST searches, applying a ≥ 99% sequence identity threshold as the criterion for assignment.

To detect and characterize a broader range of trypanosomatid species, a ~ 560 bp fragment of the 18 S ribosomal DNA gene was amplified using primers SSU561F (5′-TGG GAT AAC AAA GGA GCA-3′) and SSU561R (5′-CTG AGA CTG TAA CCT CAA AGC-3′), following the protocol described by (Noyes et al. 1996). The PCR conditions consisted of an initial denaturation at 94 °C for 3 min; followed by 35 cycles of denaturation at 94 °C for 1 min, annealing at 54 °C for 2 min, and extension at 72 °C for 1 min; with a final extension at 72 °C for 10 min.

Amplicons from all three PCR assays were resolved by electrophoresis on 2% agarose gels prepared in 1× TAE buffer, stained with RedGel (Biotium, USA), and visualized under UV light using a BioDoc-It2™ imaging system (Analytik Jena US LLC, USA). PCR products that tested positive for the 18 S rDNA gene but negative for SatDNA were submitted for Sanger sequencing at Genewiz (South Plainfield, NJ, USA).

### Bioinformatic analyses

Forward and reverse reads for the 18 S rRNA genetic marker was assembled and edited to generate consensus sequences using Geneious Prime v2025.0.2 (Kearse et al. 2012). Sequence alignments were performed using the MAFFT online server v7 (https://mafft.cbrc.jp/alignment/server/; Katoh et al. 2019) employing the Auto option, which automatically selects the most appropriate alignment algorithm (e.g., FFT‑NS‑1, FFT‑NS‑2, FFT‑NS‑i, or L‑INS‑i) based on dataset size, sequence length, and sequence divergence. Under this framework, gap opening and gap extension penalties are internally optimized by MAFFT to maximize positional homology, avoiding the need for manual parameter adjustment. The resulting alignments were manually inspected and edited in AliView v1.28 (Larsson 2014). The best-fit nucleotide substitution model was selected using jModelTest2 implemented through the CIPRES Science Gateway (https://www.phylo.org/) (Miller et al. 2010).

Phylogenetic relationships among *Trypanosoma* sequences derived from the 18 S rRNA marker were inferred using Bayesian Inference (BI) and maximum likelihood (ML) analysis in Geneious Prime v2025.0.2 and the IQ-TREE web server (http://iqtree.cibiv.univie.ac.at/) (Trifinopoulos et al. 2016), respectively. The general time-reversible model with gamma-distributed rate and invariant sites (GTR + G + I) was used as the evolutionary model, with *T. cruzi* Silvio 10X (AF303659) as outgroup for both analyses. For BI, Markov Chain Monte Carlo (MCMC) analyses were run for two million generations, with trees sampled every 100 generations. Posterior probabilities were estimated from the sampled trees after discarding the first 25% as burn-in to ensure convergence and stationarity. ML node support was assessed using non-parametric bootstrapping with 1,000 replicates. Posterior probabilities (PP) ≥ 0.70 and Bootstrap values (BS) ≥ 70% were considered to indicate strong clade support. Final phylogenetic trees were visualized and edited using FigTree v1.4.4v.

## Results

### Mammal and vector hosts of *Trypanosoma* spp.

A total of 165 adult *T. dimidiata* (*sensu lato*) individuals were collected during the study period. Both sexes were present in similar proportions across sampling years (females = 82; males = 83), no nymphs were collected. Among mammals, we captured 171 bats and 103 rodents. The bat assemblage included 12 species from four families: Phyllostomidae, Molossidae, Mormoopidae, and Vespertilionidae. The most abundant and consistently recorded species were *Pteronotus parnellii* (Mormoopidae) and *Artibeus jamaicensis* (Phyllostomidae). Rodents comprised five species from the families Cricetidae and Heteromyidae. *Heteromys gaumeri* (Heteromyidae) was the most frequently captured species, followed by *Ototylomys phyllotis* (Cricetidae) (Table 1).

**Table 1:** Vector and host species collected in the tropical forest of Kaxil Kiuic Biocultural Reserve and testing positive for Trypanosoma cruzi by SatDNA detection across sampling years. Data shown the number of positive individuals (Pos) over the total analyzed individuals (N)

	Species	Year											
		2014		2015		2016		2017		2018		2019	
Vector		Female	Male	Female	Male	Female	Male	Female	Male	Female	Male	Female	Male
		Pos/N	Pos/N	Pos/N	Pos/N	Pos/N	Pos/N	Pos/N	Pos/N	Pos/N	Pos/N	Pos/N	Pos/N
	Triatoma dimidiata		1/1	1/1	3/3	4/4	3/3	21/28	27/31			41/49	34/44
Host													
Chiroptera				2/3	10/17	4/8	9/21	5/15	11/40	4/10	13/15	14/24	7/18
Molossidae													
	Molossus rufus								1/1			0/1	
Mormoopidae	Mormoops megalophylla					1/1		2/2					
	Pteronotus davyi							1/1				6/7	
	Pteronotus parnellii					2/3	5/7	0/2	1/8	1/5	9/11	5/9	0/1
Phyllostomidae	Artibeus jamaicensis				8/15		2/8	1/9	4/23	3/5	4/4	3/7	6/15
	Artibeus lituratus					0/2	1/2						
	Dermanura phaeotis			1/1	1/1		0/2						1/1
	Dermanura tolteca			1/1									
	Desmodus rotundus				1/1	0/1		1/1	5/6				0/1
	Glossophaga mutica			0/1		1/1							
	Sturnira parvidens						1/2						
Vespertilionidae	Myotis keaysi								0/2				
Rodentia				3/5	2/8	15/23	2/7	8/18	1/11	7/8	2/4	5/11	3/8
Cricetidae	Oryzomys couesi										0/1	1/2	
	Otonyctomys hatti												0/1
	Ototylomys phyllotis				1/3			2/6	0/4	1/1	1/2	1/2	1/2
	Peromyscus leucopus					0/1							
Heteromydae	Heteromys gaumeri			3/5	1/5	15/22	2/7	6/12	1/7	6/7	1/1	3/7	2/5

### Detection of *T. cruzi* via SatDNA

A total of 171 bats, 103 rodents, and 165 *T. dimidiata* specimens were screened for *T. cruzi* using the SatDNA molecular marker. Among vectors, 135 of 165 individuals tested positive (67 out of 82 females and 68 out of 83 males), resulting in a molecular detection rate of 81.8% (95% confidence interval [CI]: 75.07–87.38%). In bats (Order Chiroptera), 79 of 171 individuals tested positive, with an overall prevalence of 46.2% (95% CI: 38.56–53.97%). Among rodents (Order Rodentia), 48 of 103 individuals tested positive, yielding a prevalence of 46.6% (95% CI: 36.7–56.7%).

Mormoopid bats, including *Mormoops megalophylla* and *Pteronotus davyi*, showed high positivity rates—up to 100% in some cases—but sample sizes were limited (e.g., *M. megalophylla* *n* = 3; and *P. davyi*: *n* = 8). *P. parnellii* also exhibited variable prevalence across years (e.g., 5/7 in one season, 0/2 in another), highlighting temporal fluctuation in infection or detectability.

Among frugivorous phyllostomid bats, *A. jamaicensis* was the most frequently sampled species (*n* = 86), with an overall prevalence of 36%. Positivity varied across sampling events, ranging from 11% (1/9) to 100% (4/4), indicating that infections are common but heterogeneous within this species. Hematophagous bats such as *Desmodus rotundus* also tested positive (70%; 7/10), reinforcing their relevance as potential contributors to sylvatic *T. cruzi* transmission.

Most positive detections were associated with *H. gaumeri*, which was well represented across all sampled years (*n* = 78) and showed consistently high infection rates (e.g., 15/22, 6/7) resulting in an overall prevalence of 51%. Other rodent species such as *O. phyllotis* and *Oryzomys couesi* were also found infected but were less frequently sampled (Table 1).

### Miniexon-based *T. cruzi* DTU identification

Using the Miniexon PCR assay, *T. cruzi* was detected in 42 of 171 bat samples, yielding an overall prevalence of 25.5% (95% CI: 18.3–31.7%). In rodents, 13 out of 103 individuals tested positive, with a prevalence of 12.6% (95% CI: 6.9–21.1%), while in *T. dimidiata*, 46 out of 165 specimens were positive, resulting in a prevalence of 28.7% (95% CI: 21.1–35.3%).

**Fig. 2:**
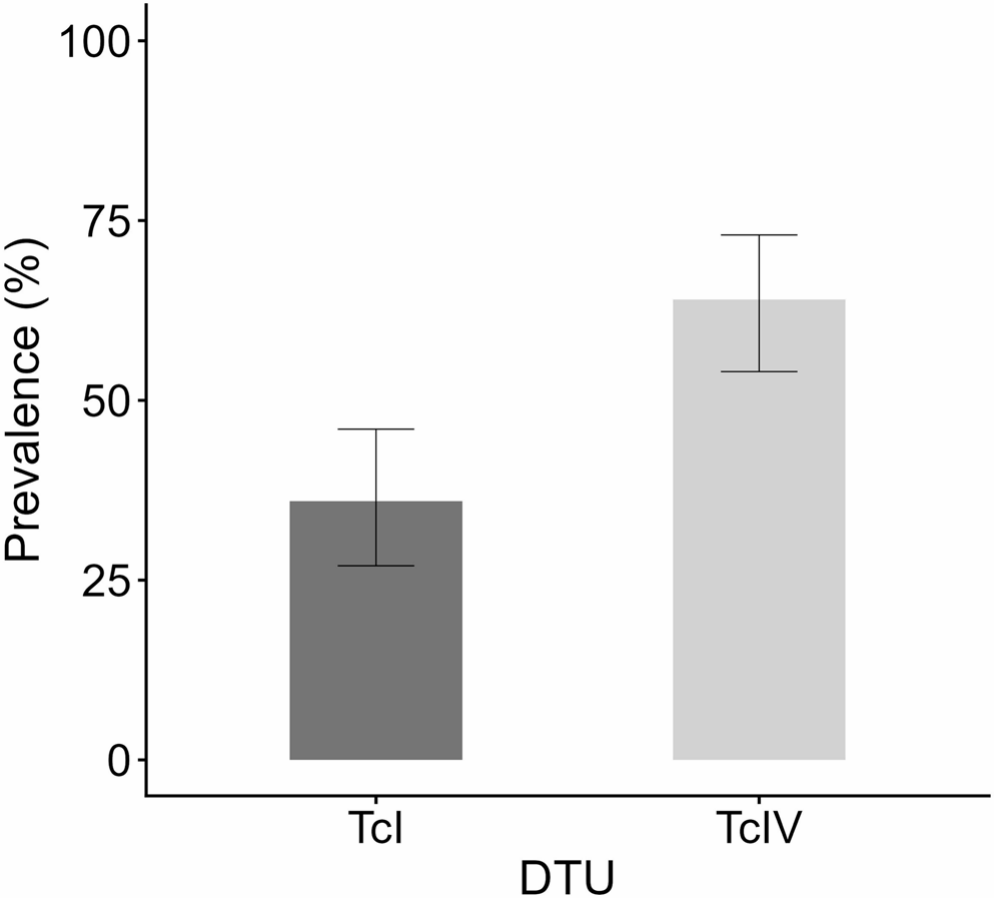
Prevalence of *Trypanosoma cruzi* discrete typing units (DTUs) TcI and TcIV across all positive hosts (vectors and mammals) in a conserved tropical dry forest region of the Yucatán Peninsula, Mexico. Bars represent the proportion of each DTU among all DTU-positive samples (*n* = 101), with 95% confidence intervals, differences between DTUs were highly significant (two-proportion z-test, Z = 4.08, *p* < 0.001)

**Fig. 3:**
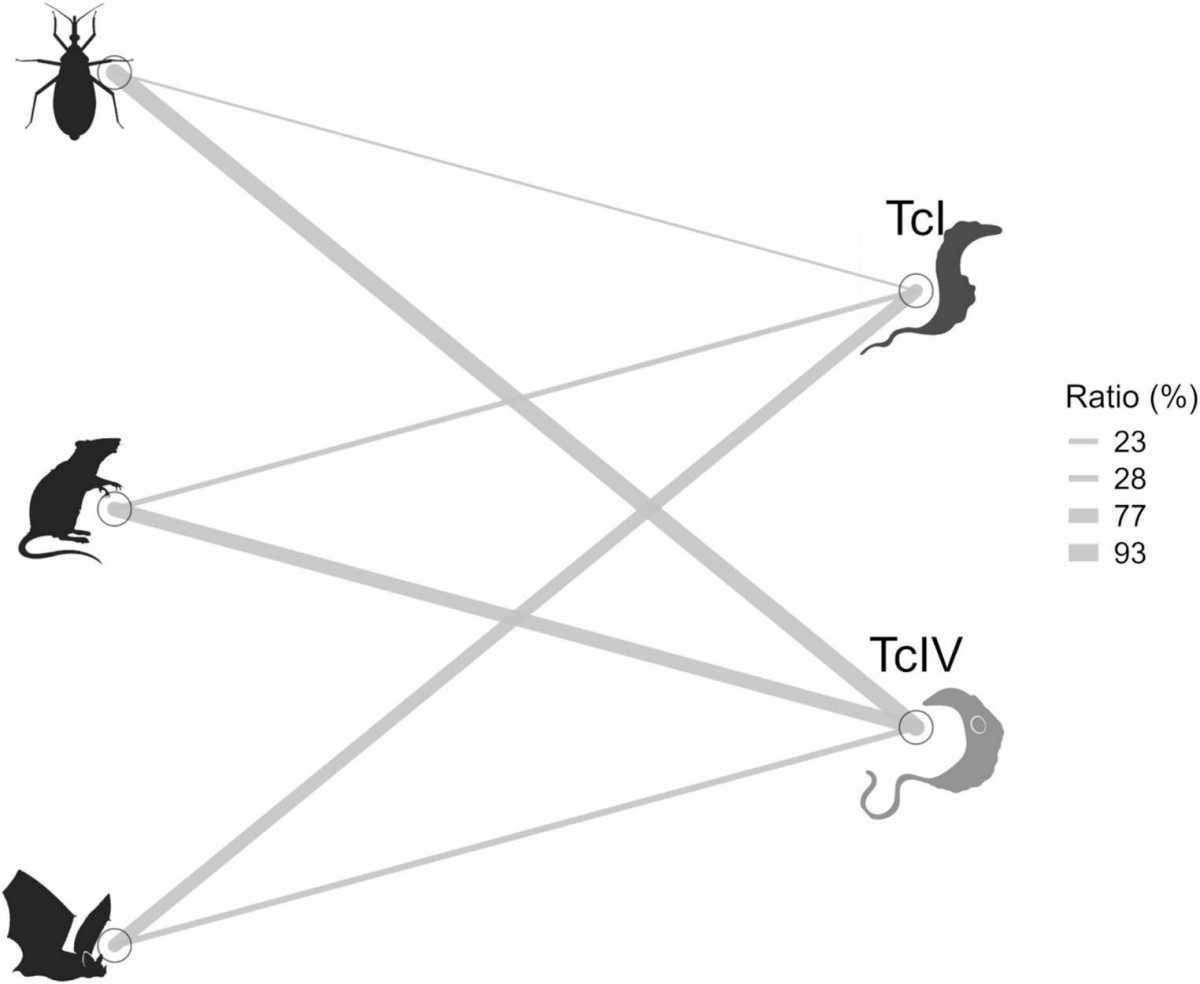
Network diagram illustrating the associations between *Trypanosoma cruzi* discrete typing units (DTUs) and host/vector groups in a conserved tropical dry forest of the Yucatán Peninsula. Edges represent detected connections between triatomine bugs, rodents, bats, and two *T. cruzi* DTUs: TcI and TcIV. Line thickness corresponds to the relative frequency of detection (ratio %) for each host–DTU pair, as indicated in the grayscale gradient

**Fig. 4:**
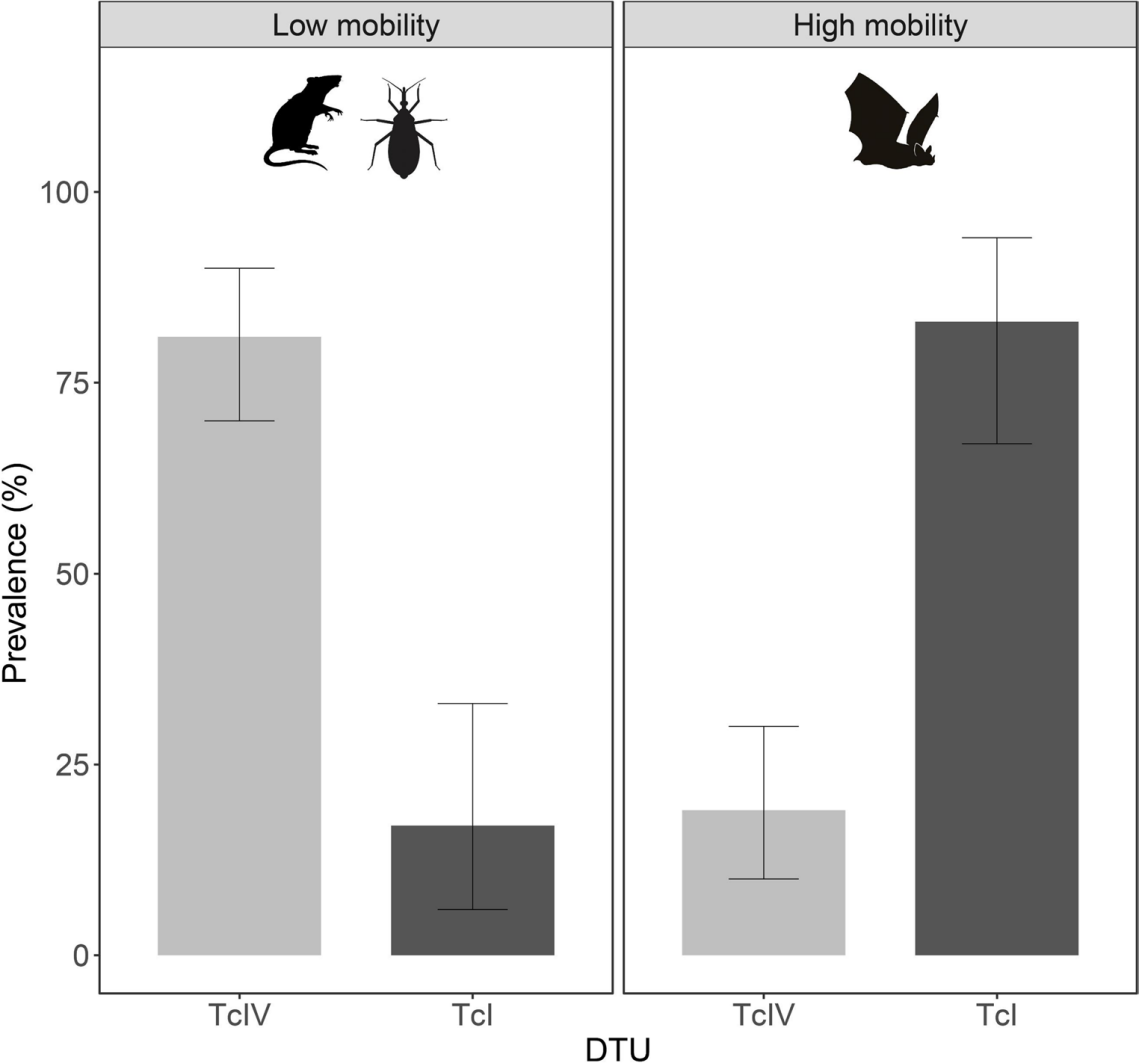
Association between *Trypanosoma cruzi* discrete typing units (DTUs) and host mobility groups in a conserved tropical dry forest region of the Yucatán Peninsula, Mexico. Bars represent the proportion of DTU-positive individuals infected with either TcI or TcIV within two host mobility categories: low mobility (rodents and triatomine vectors, left panel) and high mobility (bats, right panel). Error bars indicate 95% confidence intervals. Differences in DTU frequencies within each mobility group were statistically significant (low-mobility group: two-proportion z-test, Z = 8.82, *p* < 0.000001; high-mobility group: Z = 4.17, *p* < 0.001)

The highest prevalence with this method was recorded in 2015, with 5 of 20 individuals testing positive (25.0%), whereas in 2016, only 1 of 28 individuals tested positive (4.0%). Across all vectors and mammalian hosts combined (*T. dimidiata*, bats and rodents), TcIV was significantly more frequent than TcI, representing 65 of the 101 DTU‑positive samples (64.3%; 95% CI: 54.2–74%), whereas TcI accounted for 36 of 101 DTU‑positive samples (35.6%; 95% CI: 27–46%). (two-proportion z-test, Z = 4.08, *p* < 0.001; Fig. 2). Among insect vectors, 46 specimens tested positive by Miniexon PCR, with 43 harboring TcIV and 3 harboring TcI. In bats, TcIV was identified in 12 individuals and TcI in 30. In rodents, TcIV was detected in 10 individuals and TcI in 3 (Fig. 3). Vectors and mammalian hosts were reclassified into two qualitative mobility-capacity categories—low (rodents and *T. dimidiata*) and high (bats)—based primarily on morphological and life-history traits (e.g., flight capability and body size). Within the low-mobility group, TcIV (53/59; 89.8%) was significantly more frequent than TcI (6/59; 10.2%) (two-proportion z-test, Z = 8.82, *p* < 0.000001). Conversely, in the high-mobility group, TcI (30/42; 71.4%) predominated over TcIV (12/42; 28.6%) (two-proportion z-test, Z = 4.17, *p* < 0.001; Fig. 4).

**Fig. 5:**
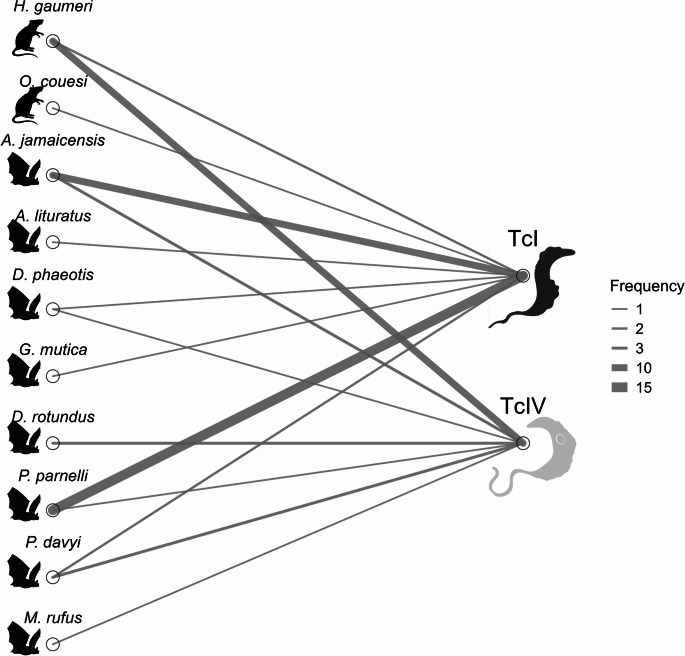
Host–parasite interaction network showing the distribution of *Trypanosoma cruzi* discrete typing units (TcI and TcIV) across mammalian host species sampled in a conserved tropical dry forest of the Yucatán Peninsula. Line thickness indicates the frequency of detection per host–DTU pair, as denoted in the legend (1–15 occurrences)

We identified host species associated with both DTUs, including *H. gaumeri* (Rodentia), which showed a stronger association with TcIV (10 out of 12 detections), and several bat species, including *A. jamaicensis*,* Dermanura phaeotis*,* P. davyi*, and *P. parnellii*, however, *A. jamaicensis* and *P. parnellii* showed a strongest association with TcI, with ten out of thirteen and fifteen out of sixteen detections respectively. *Oryzomys couesi* (Rodentia) was detected only with TcI, while *D. rotundus* (Chiroptera) was found only with TcIV (Fig. 5).

### Phylogenetic inference based on 18 S rDNA of *Trypanosoma* lineages

**Fig. 6:**
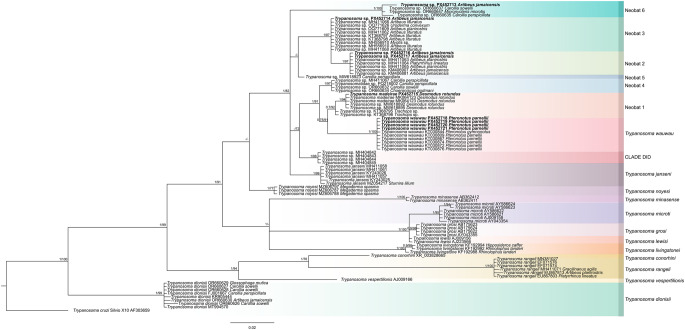
Phylogenetic relationships among representative members of the genus *Trypanosoma* based on 18 S rDNA sequences. The topology was inferred using both Bayesian inference and Maximum Likelihood analyses. Posterior probabilities (PP) from the Bayesian analysis and bootstrap support values (BS) from the Maximum Likelihood analysis are shown at the nodes. PP ≥ 0.70 and BS ≥ 70% were considered indicative of strong clade support. Each major lineage is represented by a distinct color. *Trypanosoma cruzi* Silvio 10X (AF303659) was used as the outgroup. Sequences generated in this study are shown in bold, and GenBank accession numbers for these new sequences are provided

Phylogenetic analysis of 18 S rDNA sequences from bat samples collected in the KKBR revealed a previously underappreciated diversity of *Trypanosoma* species circulating in this sylvatic environment. Sequences obtained from this study were aligned with reference data from GenBank and analyzed in a comparative phylogenetic framework that included representative lineages from Latin America and other global regions (Fig. 6).

Sequences obtained from *P. parnellii* (PX452718, PX452719, PX452720, and PX452721) formed a strongly supported monophyletic clade (posterior probability [PP] = 1; bootstrap support [BS] = 100) closely related to *T. wauwau*. Sequences from *A. jamaicensis* clustered within the Neobat 2 (PX452716 and PX452717; PP = 1; BS = 97), Neobat 3 (PX452714; PP = 1; BS = 87), and Neobat 6 (PX452713; PP = 1; BS = 100) clades. In addition, a sequence from the hematophagous bat *D. rotundus* (PX452715) grouped within the *T. madeirae* (Neobat 1) clade (PP = 1; BS = 94).

We detected three bats coinfected with *T. cruzi* and other bat-associated *Trypanosoma* species. The *D. rotundus* individual harboring Neobat 1 (*T. madeirae*) was also positive for *T. cruzi* by SatDNA PCR. Similarly, two *P. parnellii* individuals that clustered with *T. wauwau* tested positive for *T. cruzi* by SatDNA; one of these was also positive by Miniexon PCR and was assigned to TcI.

## Discussion

This study provides a comprehensive and novel perspective on *Trypanosoma* spp. diversity and persistence in a conserved tropical dry forest of the Yucatán Peninsula. By simultaneously examining triatomine vectors, bats, and rodents using complementary molecular detection and phylogenetic approaches, we demonstrate the continued circulation of *T. cruzi* DTUs TcI and TcIV in sylvatic environments. In addition, we report, for the first time in Mexico, molecular evidence of bat-associated trypanosomes closely related to *T. wauwau*, *T. madeirae* (Neobat 1), and the Neobat 2 and Neobat 3 lineages. Although Neobat 6 has been previously reported in Mexico (Juárez-Gabriel et al. 2024), this is the first record of this lineage in the Yucatán Peninsula. Notably, we also document for the first time coinfections involving *T. madeirae* (Neobat 1) and *T. cruzi* (DTU not identified), as well as *T. wauwau* with *T. cruzi* DTU TcI. Together, these findings substantially expand the known biogeographic distribution of bat-associated *Trypanosoma* lineages, which remain historically under documented in Mesoamerican dry tropical ecosystems, and underscore the ecological complexity of *Trypanosoma*–host interactions in conserved sylvatic landscapes.

While prior work in the region has confirmed the presence of *T. cruzi* in bats and small mammals (e.g., *A. jamaicensis* and *H. gaumeri*), most studies have focused on peridomestic or fragmented landscapes (López-Cancino et al. 2015; Moo-Millán et al. 2024). In contrast, our study provides evidence of the co-occurrence of *T. cruzi* in both small mammal hosts and their insect vectors within a well-preserved sylvatic environment. We found a high prevalence of infection in vectors (81.8%) and substantial infection levels in chiropteran and rodent hosts. Although we did not perform blood meal analyses for *T. dimidiata* in this study, previous work in nearby regions (Dumonteil et al. 2018; Moo-Millan et al. 2019) has identified rodents and bats as blood sources for this vector species. These findings suggest the potential for a sylvatic transmission network involving these hosts, although further studies—particularly on vector feeding patterns—are necessary to confirm direct transmission links and the stability of the transmission cycle.

Our findings confirm the widespread circulation of DTU TcI across *T. dimidiata* (s. l.), rodents, and bats in a mature tropical dry forest ecosystem. This DTU was predominantly found in bats, which have greater mobility capacity compared to the other host groups assessed. This aligns with prior studies indicating that volant mammals can traverse several kilometers across landscapes due to flight (Richardson et al. 2021), potentially facilitating broader dissemination of *T. cruzi* lineages. In contrast, DTU TcIV was found particularly in rodents and triatomine vectors—groups characterized by more limited movement. Rodents typically disperse over short distances (often hundreds of meters), with rare long-range movements (Kauer et al. 2025) T. *dimidiata* has also been shown to exhibit low mobility, with dispersal distances below 100 m over short time periods (Barbu et al. 2010). Based on these well-established differences in dispersal capacity, we hypothesize that TcIV may be more spatially constrained and particularly associated with sylvatic transmission cycles in lower-mobility hosts, as has also been reported in other studies from Mexico (Díaz-Valdez et al. 2021).

DTU TcI has long been recognized as the dominant genetic lineage of *T. cruzi* in Mesoamerica (Zingales et al. 2012; Brenière et al. 2016; Izeta-Alberdi et al. 2016), whereas TcIV appears to have a more restricted ecological niche. Most prior studies in the region have focused on peridomestic settings or degraded landscapes, where DTU structure is often obscured by overlapping host and vector ecotopes (López-Cancino et al. 2015; Moo-Millan et al. 2019). By contrast, our study provides rare evidence of TcIV maintenance in sylvatic reservoirs, including *H. gaumeri*, *A. jamaicensis*, *D. phaeotis*, *D. rotundus*, *P. parnellii* and *P. davyi*—species known for their ecological ubiquity but rarely evaluated together in well-conserved habitats. The high infection prevalence observed in *T. dimidiata* and small mammals (~ 46%) strongly supports the view that sylvatic transmission cycles are independently sustained in this ecosystem and not merely the result of anthropogenic spillback. Moreover, the co‑detection of TcI across multiple host taxa and sampling periods suggests long‑term stability of the sylvatic transmission network. This pattern is consistent with metacommunity frameworks in which generalist vectors, such as *T. dimidiata*, connect multiple host species across ecological guilds (Ibarra‑Cerdeña et al. 2017; Hodo and Hamer 2017). *T. dimidiata* is considered a generalist in ecological terms because it feeds on a wide diversity of vertebrate hosts, including rodents, bats, domestic animals, and humans, across sylvatic, peridomestic, and domestic habitats. Blood‑meal analyses using metabarcoding and next‑generation sequencing in the Yucatán Peninsula have demonstrated that *T. dimidiata* (s. l.) exploits highly diverse host assemblages and frequently switches among host species (Dumonteil et al. 2018; Moo‑Millán et al. 2019). This broad host use likely facilitates the circulation of TcI among taxa with contrasting ecologies and dispersal capacities, contributing to the persistence of transmission networks within gradient of conserved-anthropized landscape. In this context, bats and rodents may act not only as potential reservoirs but also as complementary hosts that contribute to the persistence of *T. cruzi* across space and time.

Our phylogenetic analyses based on the 18 S rRNA gene revealed several bat *Trypanosoma* lineages. Sequences obtained from *A. jamaicensis* formed well-supported monophyletic groups clustered with known Neobat clades (e.g., Neobat 2, 3, and 6). Notably, sequences obtained from *P. parnellii* clustered with *T. wauwau*, a bat-restricted *Trypanosoma* species previously reported only in *Pteronotus* spp. from South America and Guatemala (Lima et al. 2015a), thereby extending its known northern distribution into Mexico. Additionally, we detected the presence of the *T. madeirae* (Neobat 1) lineage in the common vampire bat, *D. rotundus*. This *Trypanosoma* species was recently described from vampire bats in Brazil (Barros et al. 2019). To our knowledge, this represents the first record of this lineage in Mesoamerica. These findings highlight the underestimated diversity of bat-associated *Trypanosoma* in Mesoamerica and support the hypothesis that bat hosts in tropical dry forests constitute a key evolutionary reservoir within the *T. cruzi* clade, consistent with the bat-seeding hypothesis (Ramírez et al. 2014; Lima et al. 2015b). Together, these results underscore the ecological and evolutionary complexity of the *T. cruzi* clade in Neotropical bat communities and emphasize the role of conserved forest habitats as reservoirs of genetically diverse and potentially novel *Trypanosoma* species.

Such findings lend further support to the “bat-seeding hypothesis,” which posits that the *T. cruzi* clade originated from ancestral bat trypanosomes and diversified via host switching and ecological adaptation (Hamilton et al. 2012; Ramírez et al. 2014). The phylogenetic positioning of our sequences—often as sister groups to known lineages but separated by significant branch lengths—suggests that many of the trypanosomes circulating in bats from Mesoamerica remain undescribed and may represent novel evolutionary lineages. These patterns are consistent with studies from South America and Africa showing that bat assemblages harbor unusually high *Trypanosoma* diversity (Cottontail et al. 2009; Lima et al. 2015a), likely reflecting their ecological diversity, mobility, and long evolutionary histories. Importantly, our study demonstrates that this hidden diversity also extends to dry forest ecosystems of Mexico, underscoring the need to incorporate under-sampled regions and hosts into future parasite systematics and surveillance efforts.

The detection of high *Trypanosoma* prevalence and unexpected lineage diversity in a well-preserved tropical dry forest has important implications for both ecological understanding and disease surveillance in the region. While sylvatic cycles of *T. cruzi* have been traditionally viewed as ecologically contained, our results suggest that mature forest ecosystems may act as long-term reservoirs of both genetic and ecological parasite diversity, including lineages with unknown zoonotic potential. This is particularly relevant in the context of ongoing land-use change and fragmentation in the Yucatan Peninsula, where deforestation by agricultural expansion, fires, and megaprojects among the main causes, are encroaching upon remnant dry forest patches (Mascorro et al. 2016; Ellis et al. 2017; Zambrano et al. 2025). Such landscape alterations can increase contact rates between humans, domestic animals, and sylvatic reservoirs, thereby intensifying the risk of spillover from cryptic transmission cycles previously restricted to forest interiors (Ramsey et al. 2012; Gottdenker et al. 2014; López-Cancino et al. 2015; Moo-Millán et al. 2019). Moreover, our study reinforces the role of bats as reservoirs of diverse trypanosomatids, in line with their importance in global zoonotic emergence frameworks (Ramírez et al. 2014; Austen and Barbosa 2021). Although most of the lineages we detected appear restricted to bat hosts, their phylogenetic proximity to zoonotic trypanosomatid species such as *T. cruzi* highlights the need for expanded surveillance of sylvatic parasites beyond known vectors and reservoirs. The frequent detection of *T. cruzi* in the vector *T. dimidiata*, a plastic species capable of inhabiting both sylvatic and domestic ecotopes, further emphasizes this risk (Montes De Oca-Aguilar et al. 2022; Moo-Millán et al. 2023).

These findings reinforce the growing consensus that landscape-level, integrative approaches are essential for effective parasitological research and public health preparedness, particularly in biodiversity-rich regions. Conserved habitats, such as the tropical dry forests of Mesoamerica, are often seen solely as refuges for ecosystem resilience; however, they may also serve as cryptic reservoirs of pathogen diversity and evolutionary novelty, including lineages with zoonotic potential (Woolhouse et al. 2012; Guégan et al. 2020). The detection of *Trypanosoma* lineages such as *T. wauwau*, *T. madeirae*, and multiple Neobat clades within protected ecosystems highlights the crucial role of these areas as natural observatories for monitoring sylvatic transmission cycles, providing early insight into pathogen dynamics before potential spillover into human-dominated environments (Austen and Barbosa 2021; Espinal-Palomino et al. 2025; Matiz-González et al. 2025). As Artois et al. (2009) and Glidden et al. (2021) emphasize, proactive surveillance in ecologically intact landscapes is central to the early detection of emerging pathogens and should be prioritized in global health strategies. Importantly, as noted by Cunningham et al. (2017) and Wang et al. (2023), effective early-warning systems must integrate ecological context with molecular data to anticipate spillover events. Our results contribute to this vision, demonstrating how conservation and disease surveillance can converge to support both biodiversity and planetary health goals.

This work contributes valuable baseline data for future research and supports the integration of sylvatic transmission dynamics into regional surveillance and One Health frameworks, particularly in ecologically sensitive landscapes undergoing rapid change.

In this study, we implemented a tiered molecular strategy to detect and characterize *Trypanosoma* infections across diverse host taxa. Although we did not aim to directly compare detection rates between these molecular markers, we acknowledge their differences in sensitivity and specificity, which reflect their distinct diagnostic roles within our workflow. Differences in detection performance likely stem from underlying variation in parasite load, infection stage, tissue tropism, and target specificity (Izeta-Alberdi et al. 2023). Our results align with previous findings that SatDNA is among the most sensitive markers for detecting active *T. cruzi* infections in wildlife. In contrast, the Miniexon marker, though less sensitive, provided critical genotyping information by allowing DTU-level identification. The use of 18 S rDNA further expanded detection capacity to non-*T. cruzi* species, underscoring its value for broader parasitological surveillance. The use of this combined molecular strategy allowed us to detect coinfections involving *T. madeirae* (Neobat 1) identified by 18 S rDNA and *T. cruzi* detected by SatDNA, as well as coinfections between *T. wauwau* identified by 18 S rDNA and *T. cruzi* DTU I identified by Miniexon PCR. These findings emphasize the importance of integrating complementary molecular tools to improve detection accuracy and to capture the diversity of co-circulating *Trypanosoma* species. Such multi-marker approaches are particularly critical in sylvatic settings, where ecological complexity and high host diversity can obscure parasite transmission dynamics.

Future research in this system would benefit from deeper and more integrative analyses of biological samples, combining classical parasitological approaches with expanded molecular tools, including multilocus genotyping and next-generation sequencing strategies such as metabarcoding. Metabarcoding approaches, in particular, can enable fine-scale taxonomic characterization of *Trypanosoma* lineages and substantially increase the ability to detect mixed infections and cryptic diversity that may remain undetected using single-marker assays (Hernández-Andrade et al. 2019).

## Supplementary Information

Below is the link to the electronic supplementary material.

Supplementary Material 1. (DOCX 14.2 KB)

Supplementary Material 2. (DOCX 15.2 MB)

## Data Availability

All relevant data are included within the manuscript and/or its supplementary files.
