# Supplementary material for: Bat-associated Trypanosoma diversity, geographic extension of known clades, and predominance of T. cruzi TcIV in a conserved tropical forest of southeastern Mexico
Source: Parasitol Res. 2026 Apr 10;125(1):60. doi: 10.1007/s00436-026-08670-w (PMC13180769; doi:10.1007/s00436-026-08670-w)
Supplement: Supplementary file 1 — Supplementary Material 1. (DOCX 14.2 KB) [file 436_2026_8670_MOESM1_ESM.docx]

Table S1. Sampling effort across years and taxonomic groups.

Summary of field sampling effort and capture success for rodents, bats, and insect vectors (Triatoma dimidiata) between 2014 and 2019 in the tropical forest of the Yucatán Peninsula, Mexico. For rodents, the number of Sherman traps used, trap-nights, and individuals captured are reported. For bats, sampling effort is expressed as the total meters of mist nets deployed per night and the number of individuals captured. For insect vectors, sampling effort is presented as total hours of light-trap operation per night and the number of triatomines collected. Capture values reflect the number of individuals retained for molecular analysis. No rodent or bat sampling occurred in 2014.

| Year | Rodents | | | Bats | | | Insect vectors | | |
| --- | --- | --- | --- | --- | --- | --- | --- | --- | --- |
|  | Sherman traps | Nights | Captured individuals | Mist net meters | Nights | Captured individuals | Hours per light trap | Nights | Captured individuals |
| 2014 | ⏤ | ⏤ | ⏤ | ⏤ | ⏤ | ⏤ | 4 | 1 | 1 |
| 2015 | 60 | 7 | 13 | 540 | 7 | 20 | 4 | 5 | 4 |
| 2016 | 80 | 4 | 30 | 621 | 5 | 29 | 4 | 3 | 7 |
| 2017 | 80 | 4 | 29 | 648 | 6 | 55 | 4 | 8 | 28 |
| 2018 | 60 | 4 | 12 | 216 | 2 | 25 | 4 | 1 | 25 |
| 2019 | 90 | 6 | 19 | 126 | 2 | 42 | 4 | 14 | 93 |
