# Supplementary material for: Bat-associated Trypanosoma diversity, geographic extension of known clades, and predominance of T. cruzi TcIV in a conserved tropical forest of southeastern Mexico
Source: Parasitol Res. 2026 Apr 10;125(1):60. doi: 10.1007/s00436-026-08670-w (PMC13180769; doi:10.1007/s00436-026-08670-w)

Gel 1. Agarose gel showing PCR products obtained using the Miniexon protocol (Fernandes et al., 2001) for the detection of *Trypanosoma cruzi* in mammal samples. The expected band (~100 bp) is visible in positive samples. The table indicates the sample code and the corresponding mammal species.

| Sample code | Species |
| --- | --- |
| KK038 | *Artibeus jamaicensis* |
| KK302 | *Artibeus jamaicensis* |
| KK032 | *Artibeus jamaicensis* |
| KK042 | *Desmodus rotundus* |
| kK300 | *Pteronotus parnellii* |
| KK118 | *Glossophaga mutica* |
| KK189 | *Heteromys gaumeri* |
| KK188 | *Heteromys gaumeri* |
| KK191 | *Heteromys gaumeri* |
| KK331 | *Heteromys gaumeri* |
| KK339 | *Heteromys gaumeri* |


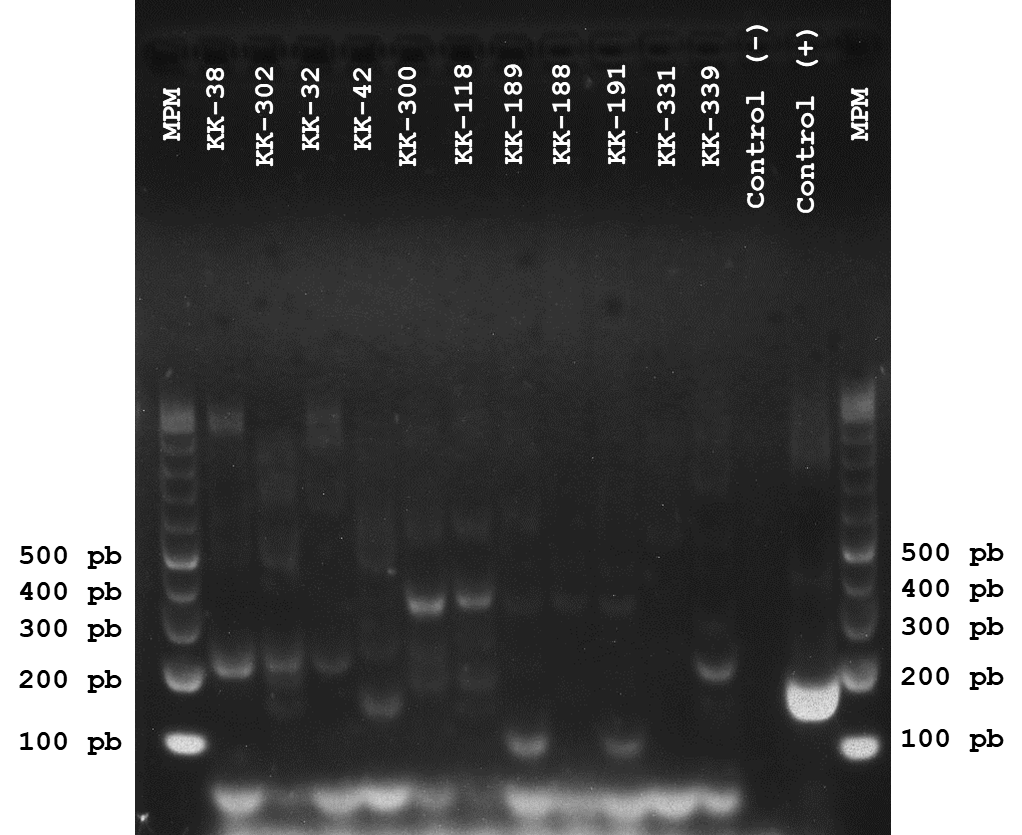


Gel 2. Agarose gel showing PCR products obtained using the Miniexon protocol (Fernandes et al., 2001) for the detection of *Trypanosoma cruzi* in mammal samples. The expected band (~100 bp) is visible in positive samples. The table indicates the sample code and the corresponding mammal species.

| KK340 | *Heteromys gaumeri* |
| --- | --- |
| KK333 | *Ototylomys phyllotis* |
| KK186 | *Heteromys gaumeri* |
| KK185 | *Heteromys gaumeri* |
| KK187 | *Heteromys gaumeri* |
| KK022 | *Heteromys gaumeri* |
| KK043 | *Heteromys gaumeri* |
| KK052 | *Ototylomys phyllotis* |
| KK063 | *Heteromys gaumeri* |
| KK064 | *Heteromys gaumeri* |
| KK276 | *Pteronotus parnellii* |
| KK165 | *Heteromys gaumeri* |
| KK224 | *Desmodus rotundus* |


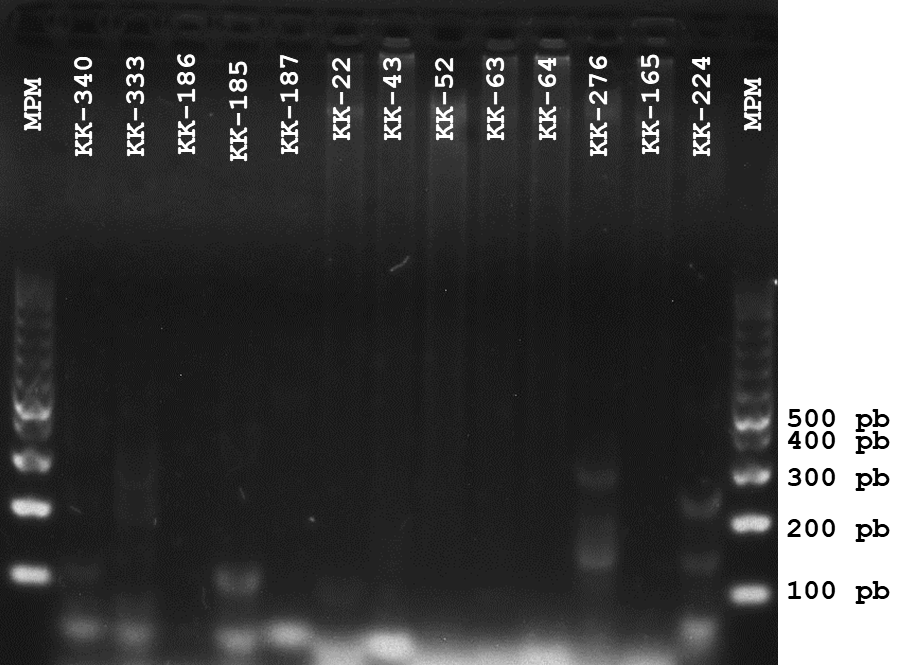


Gel 3. Agarose gel showing PCR products obtained using the Miniexon protocol (Fernandes et al., 2001) for the detection of *Trypanosoma cruzi* in mammal samples. The expected band (~100 bp) is visible in positive samples. The table indicates the sample code and the corresponding mammal species.

| KK162 | *Heteromys gaumeri* |
| --- | --- |
| KK216 | *Desmodus rotundus* |
| KK205 | *Artibeus jamaicensis* |
| KK275 | *Mormoops megalophylla* |
| KK160 | *Heteromys gaumeri* |
| KK253 | *Heteromys gaumeri* |
| KK214 | *Molossus rufus* |
| KK252 | *Heteromys gaumeri* |
| KK283 | *Ototylomys phyllotis* |
| KK222 | *Artibeus jamaicensis* |
| KK282 | *Heteromys gaumeri* |
| KK159 | *Heteromys gaumeri* |
| KK228 | *Desmodus rotundus* |


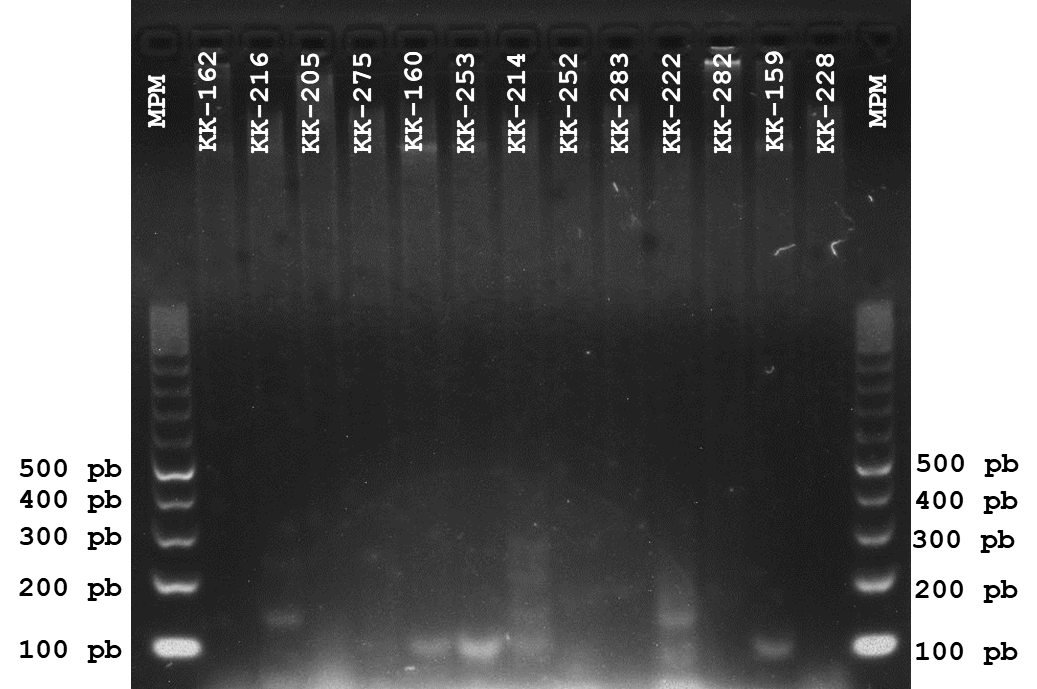


Gel 4. Agarose gel showing PCR products obtained using the Miniexon protocol (Fernandes et al., 2001) for the detection of *Trypanosoma cruzi* in mammal samples. The expected band (~100 bp) is visible in positive samples. The table indicates the sample code and the corresponding mammal species.

| KK284 | *Heteromys gaumeri* |
| --- | --- |
| KK410 | *Pteronotus davyi* |
| KK245 | *Heteromys gaumeri* |
| KK247 | *Heteromys gaumeri* |
| KK223 | *Desmodus rotundus* |
| KK195 | *Desmodus rotundus* |
| KK235 | *Artibeus jamaicensis* |
| KK264 | *Heteromys gaumeri* |
| KK277 | *Mormoops megalophylla* |
| KK350 | *Artibeus jamaicensis* |
| KK348 | *Artibeus jamaicensis* |
| KK363 | *Artibeus jamaicensis* |
| KK380 | *Heteromys gaumeri* |


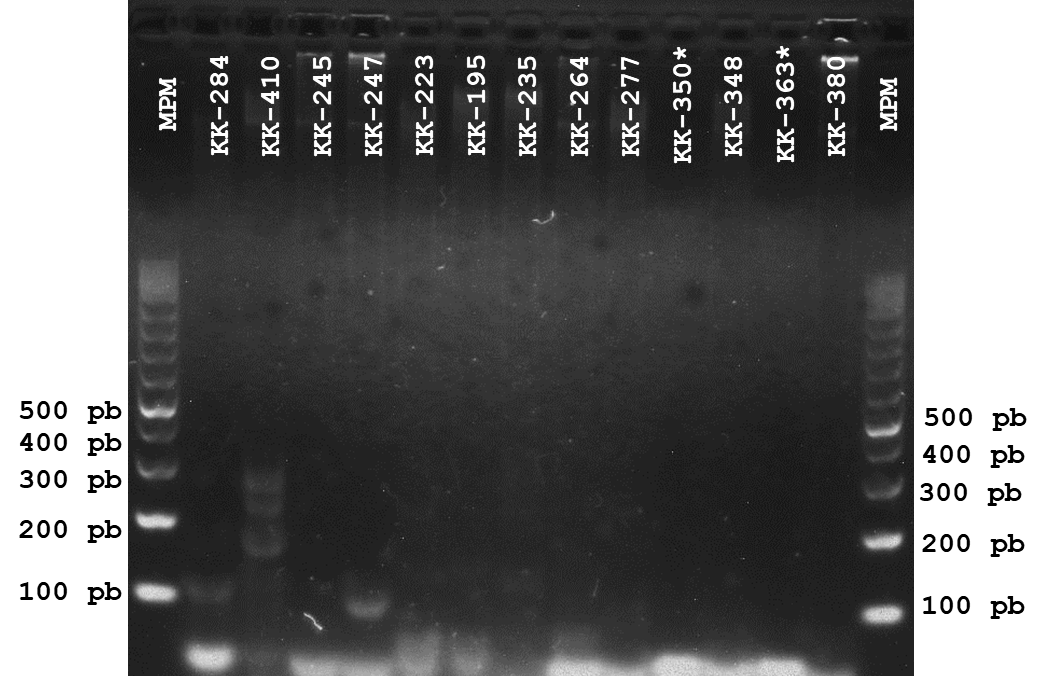


Gel 5. Agarose gel showing PCR products obtained using the Miniexon protocol (Fernandes et al., 2001) for the detection of *Trypanosoma cruzi* in mammal samples. The expected band (~100 bp) is visible in positive samples. The table indicates the sample code and the corresponding mammal species.

| KK365 | *Artibeus jamaicensis* |
| --- | --- |
| KK366 | *Pteronotus parnellii* |
| KK367 | *Artibeus jamaicensis* |
| KK368 | *Artibeus jamaicensis* |
| KK374 | *Artibeus jamaiscensis* |
| KK371 | *Dermanura phaeotis* |
| KK405 | *Pteronotus davyi* |
| KK377 | *Heteromys gaumeri* |
| KK396 | *Pteronotus parnellii* |
| KK400 | *Pteronotus davyi* |
| KK391 | *Oryzomys couesi* |
| KK392 | *Ototylomys phyllotis* |
| KK379 | *Heteromys gaumeri* |


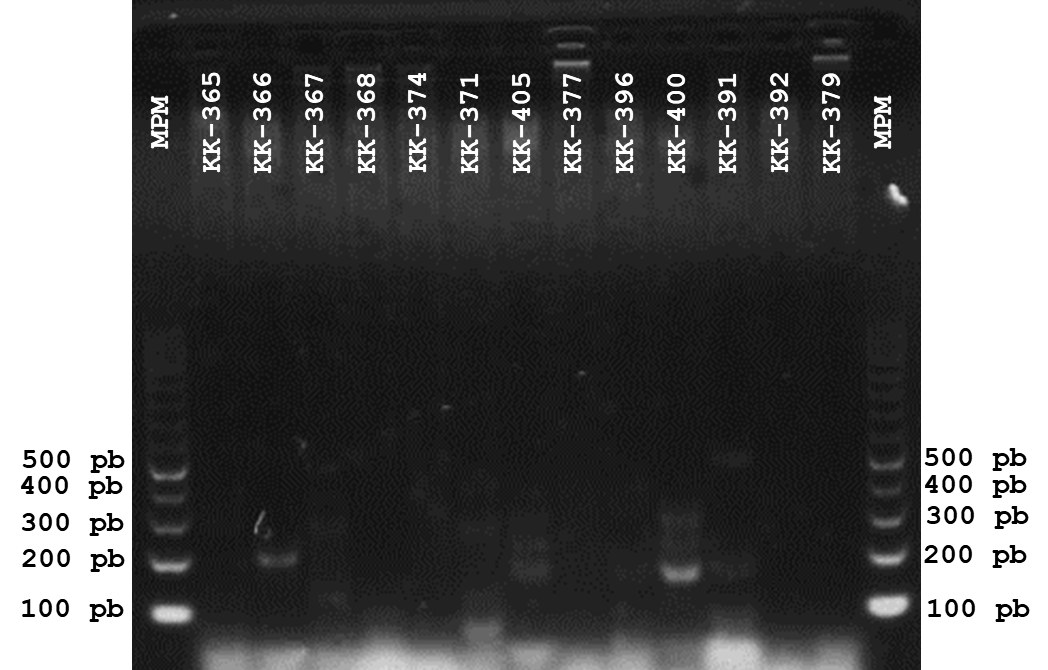


Gel 6. Agarose gel showing PCR products obtained using the Miniexon protocol (Fernandes et al., 2001) for the detection of *Trypanosoma cruzi* in mammal samples. The expected band (~100 bp) is visible in positive samples. The table indicates the sample code and the corresponding mammal species.

| KK412 | Pteronotus parnellii |
| --- | --- |
| KK383 | Heteromys gaumeri |
| KK384 | Heteromys gaumeri |
| KK414 | Pteronotus davyi |
| KK417 | Pteronotus parnellii |
| KK425 | Pteronotus parnellii |
| KK037 | Artibeus jamaiscensis |
| KK360 | Artibeus jamaicensis |
| KK029 | Artibeus jamaicensis |
| KK320 | Pteronotus parnellii |
| KK369 | Artibeus jamaicensis |
| KK399 | Pteronotus davyi |
| KK048 | Artibeus jamaicensis |


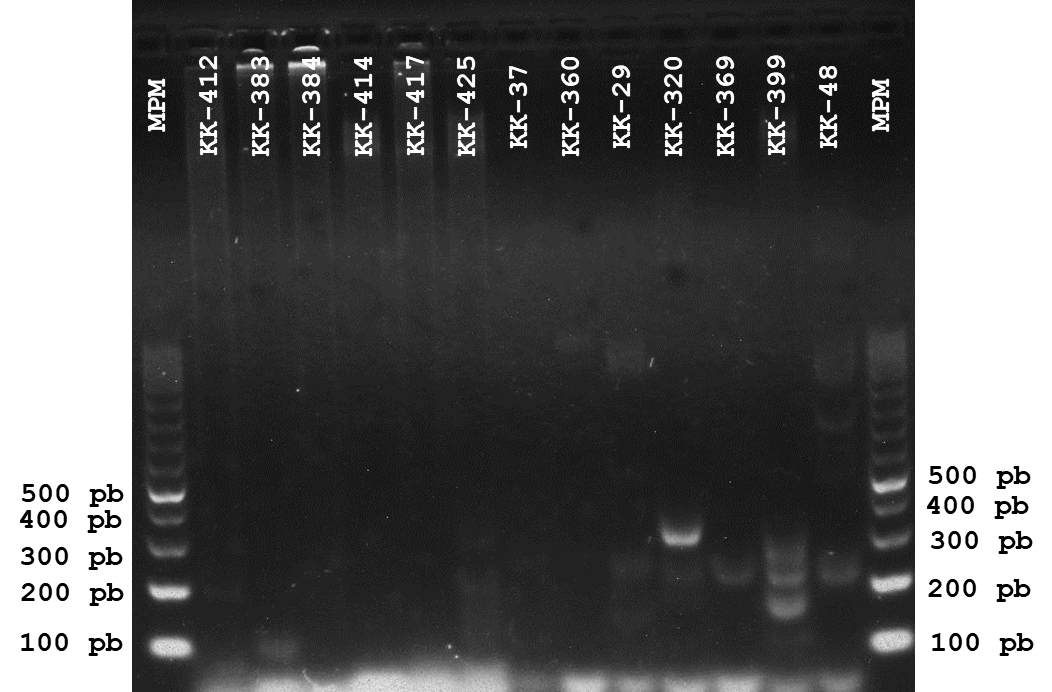


Gel 7. Agarose gel showing PCR products obtained using the Miniexon protocol (Fernandes et al., 2001) for the detection of *Trypanosoma cruzi* in mammal samples. The expected band (~100 bp) is visible in positive samples. The table indicates the sample code and the corresponding mammal species.

| KK028 | *Artibeus jamaicensis* |
| --- | --- |
| KK055 | *Dermanura tolteca* |
| KK301 | *Pteronotus parnellii* |
| KK306 | *Pteronotus parnellii* |


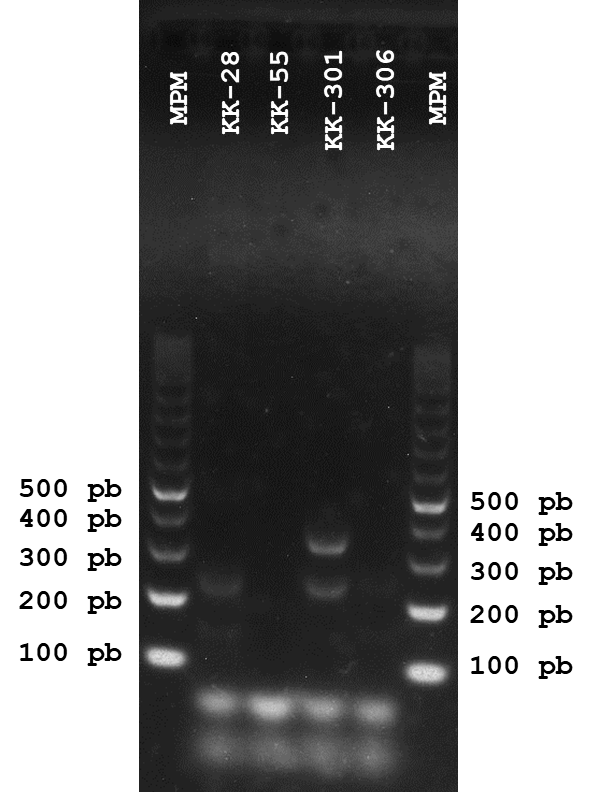


Gel 8. Agarose gel showing PCR products obtained using the Miniexon protocol (Fernandes et al., 2001) for the detection of *Trypanosoma cruzi* in mammal samples. The expected band (~100 bp) is visible in positive samples. The table indicates the sample code and the corresponding mammal species.

| KK239 | *Desmodus rotundus* |
| --- | --- |
| KK108 | *Mormoops megalophylla* |
| KK227 | *Artibeus jamaicensis* |
| KK204 | *Artibeus jamaicensis* |
| KK197 | *Pteronotus davyi* |
| KK154 | *Heteromys gaumeri* |
| KK122 | *Pteronotus parnellii* |
| KK224 | *Desmodus rotundus* |
| KK267 | *Ototylomys phyllotis* |
| KK335 | *Heteromys gaumeri* |
| KK148 | *Heteromys gaumeri* |


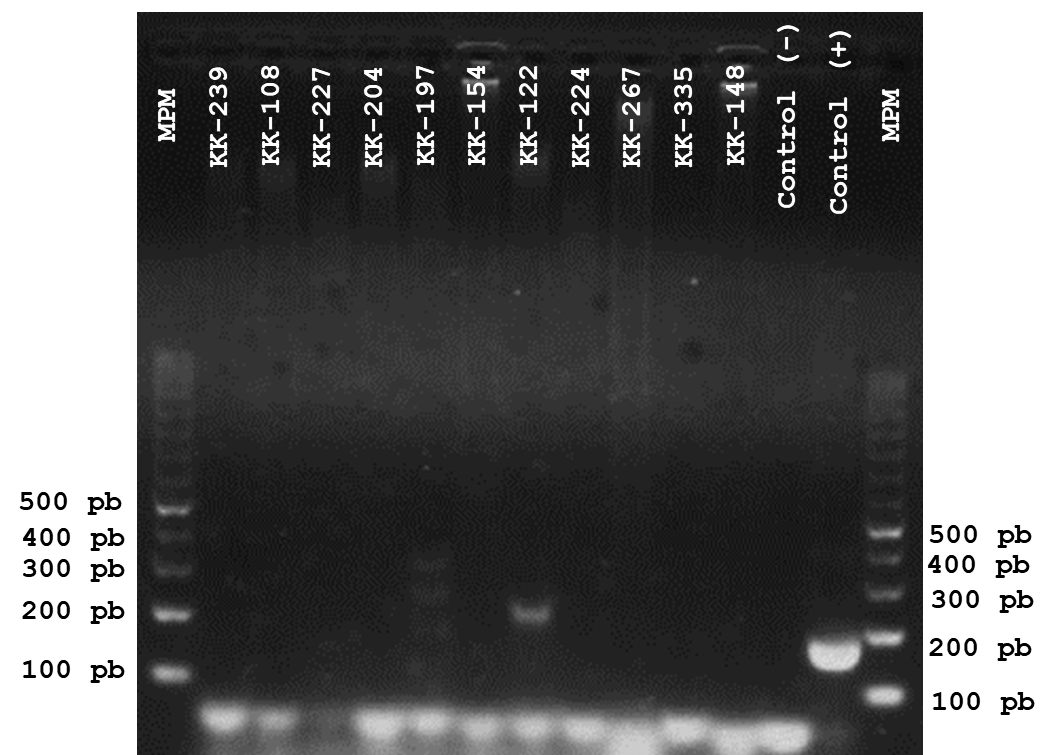


Gel 9. Agarose gel showing PCR products obtained using the Miniexon protocol (Fernandes et al., 2001) for the detection of *Trypanosoma cruzi* in mammal samples. The expected band (~100 bp) is visible in positive samples. The table indicates the sample code and the corresponding mammal species.

| KK020 | *Dermanura phaeotis* |
| --- | --- |
| KK316 | *Pteronotus parnellii* |
| KK019 | *Dermanura phaeotis* |
| KK125 | *Artibeus jamaicensis* |
| KK330 | *Heteromys gaumeri* |
| KK143 | *Pteronotus parnellii* |
| KK120 | *Artibeus lituratus* |
| KK304 | *Artibeus jamaicensis* |
| KK150 | *Heteromys gaumeri* |
| KK137 | *Sturnira parvidens* |
| KK324 | *Artibeus jamaicensis* |
| KK112 | *Pteronotus parnellii* |
| KK144 | *Pteronotus parnellii* |


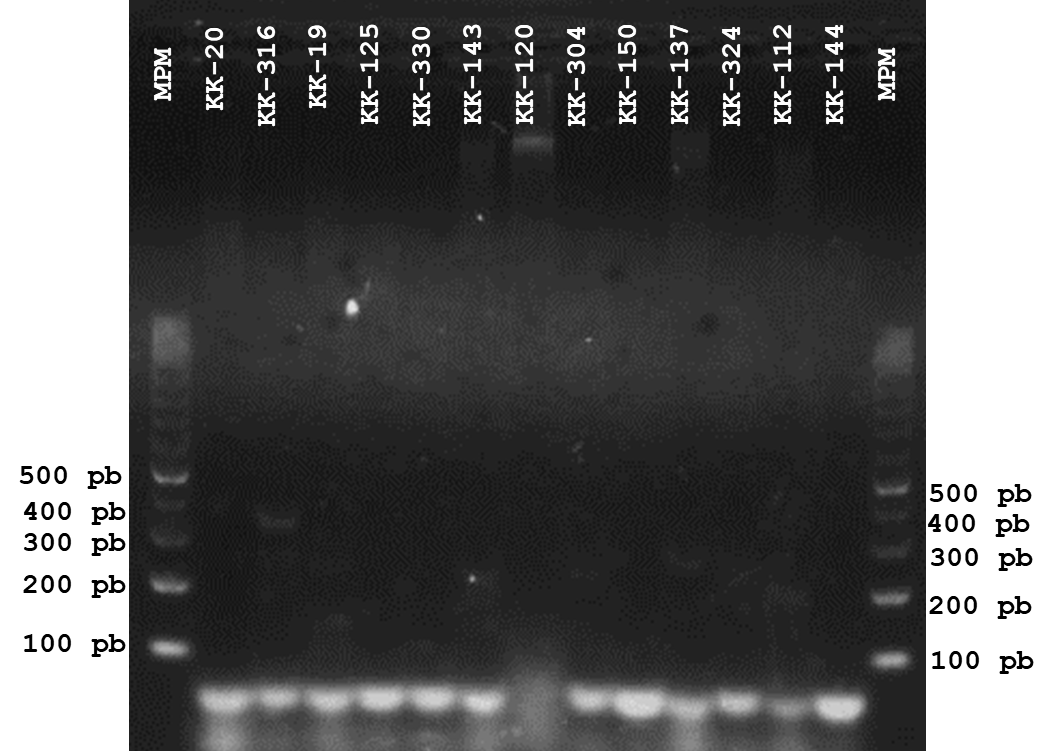


Gel 10. Agarose gel showing PCR products obtained using the Miniexon protocol (Fernandes et al., 2001) for the detection of *Trypanosoma cruzi* in mammal samples. The expected band (~100 bp) is visible in positive samples. The table indicates the sample code and the corresponding mammal species.

| KK111 | *Pteronotus parnellii* |
| --- | --- |
| KK164 | *Heteromys gaumeri* |
| KK307 | *Artibeus jamaicensis* |
| KK317 | *Artibeus jamaicensis* |
| KK157 | *Heteromys gaumeri* |
| KK113 | *Pteronotus parnellii* |
| KK306 | *Pteronotus parnelli* |
| KK141 | *Artibeus jamaicensis* |
| KK336 | *Pteronotus parnellii* |
| KK339 | *Heteromys gaumeri* |
| KK161 | *Heteromys gaumeri* |
| KK163 | *Heteromys gaumeri* |
| KK392 | *Ototylomys phyllotis* |


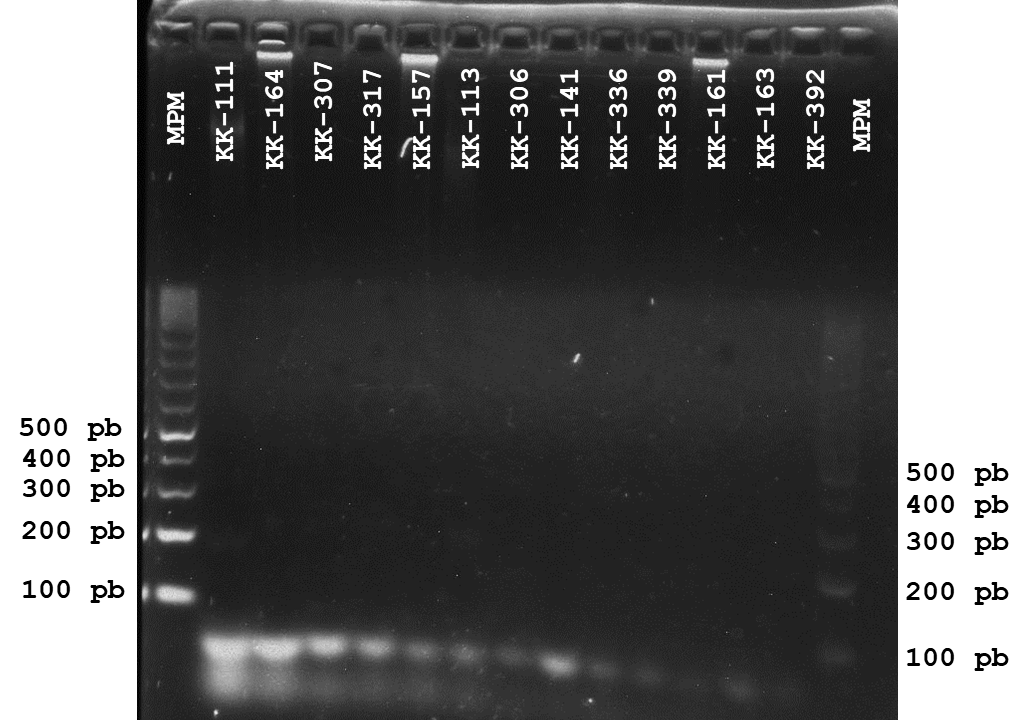


Gel 11. Agarose gel showing PCR products obtained using the Miniexon protocol (Fernandes et al., 2001) for the detection of *Trypanosoma cruzi* in mammal samples. The expected band (~100 bp) is visible in positive samples. The table indicates the sample code and the corresponding mammal species.

| KK310 | *Pteronotus parnellii* |
| --- | --- |
| KK311 | *Artibeus jamaicensis* |
| KK338 | *Heteromys gaumeri* |
| KK318 | *Pteronotus parnellii* |
| KK313 | *Artibeus jamaicensis* |
| KK323 | *Pteronotus parnellii* |
| KK117 | *Pteronotus parnellii* |
| KK318 | *Pteronotus parnellii* |
| KK233 | *Artibeus jamaicensis* |


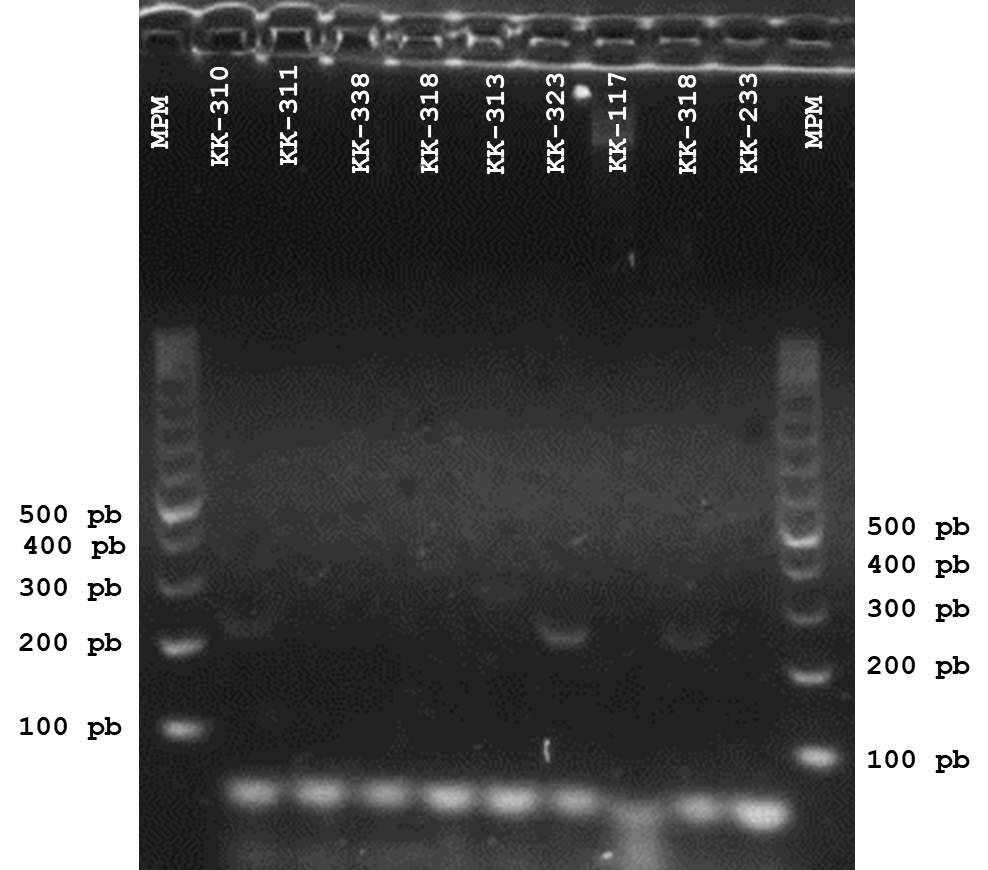


Gel 12. Agarose gel showing PCR products obtained using the Miniexon protocol (Fernandes et al., 2001) for the detection of *Trypanosoma cruzi* in *Triatoma dimidiata*. The expected band (~100 bp) is visible in positive samples. The first sample corresponds to a mammal. The remaining samples correspond to intestinal content or feces from the vector *Triatoma dimidiata*.

| KK319 | Pteronotus parnellii |
| --- | --- |


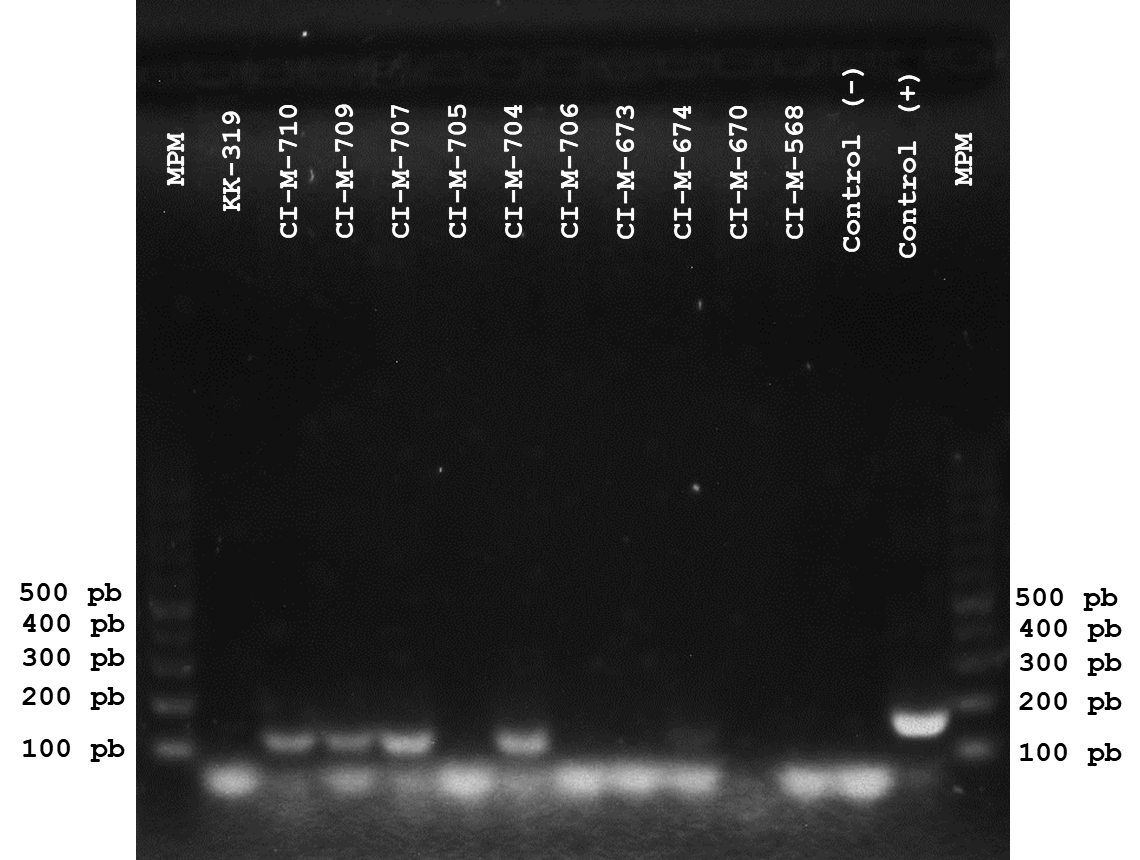


Gel 13. Agarose gel showing PCR products obtained using the Miniexon protocol (Fernandes et al., 2001) for the detection of *Trypanosoma cruzi* in *Triatoma dimidiata*. The expected band (~100 bp) is visible in positive samples. The samples correspond to intestinal content or feces


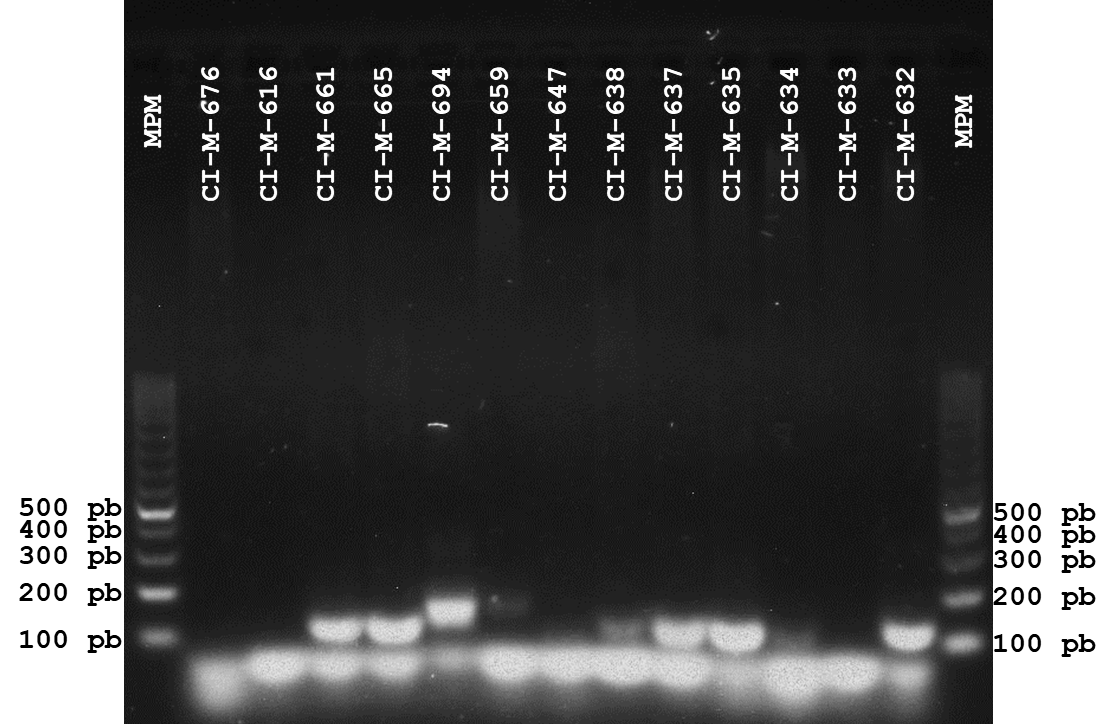


Gel 14. Agarose gel showing PCR products obtained using the Miniexon protocol (Fernandes et al., 2001) for the detection of *Trypanosoma cruzi* in *Triatoma dimidiata*. The expected band (~100 bp) is visible in positive samples. The samples correspond to intestinal content or feces.
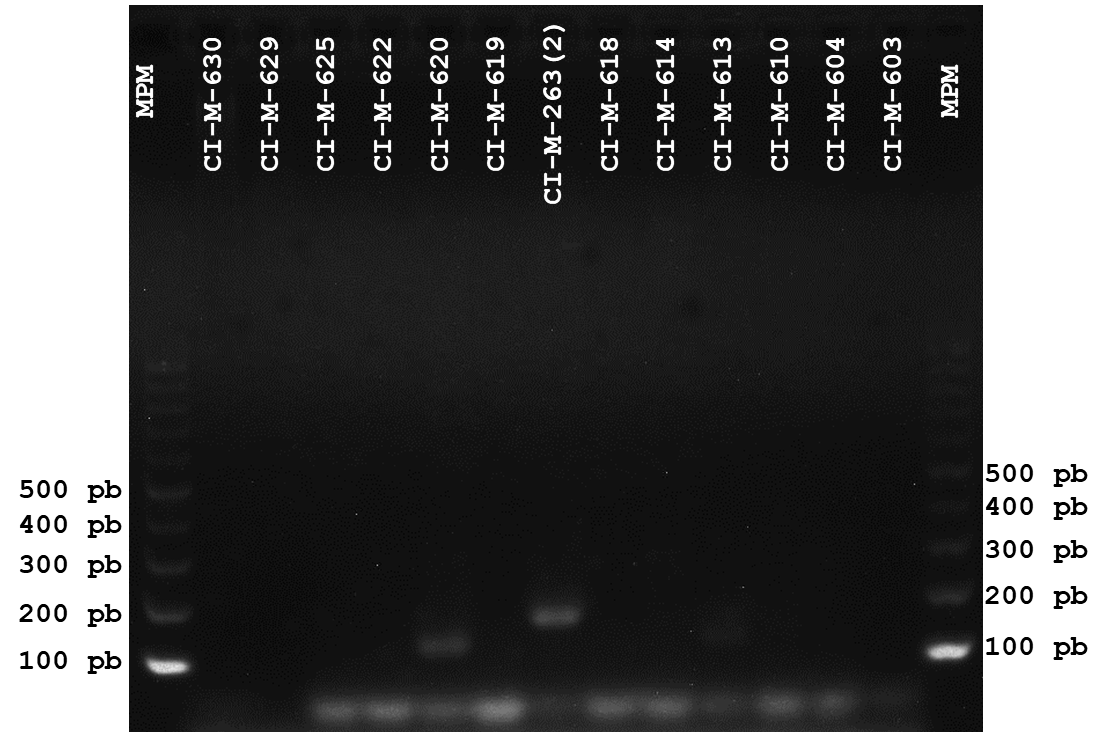


Gel 15. Agarose gel showing PCR products obtained using the Miniexon protocol (Fernandes et al., 2001) for the detection of *Trypanosoma cruzi* in *Triatoma dimidiata*. The expected band (~100 bp) is visible in positive samples. The samples correspond to intestinal content or feces.


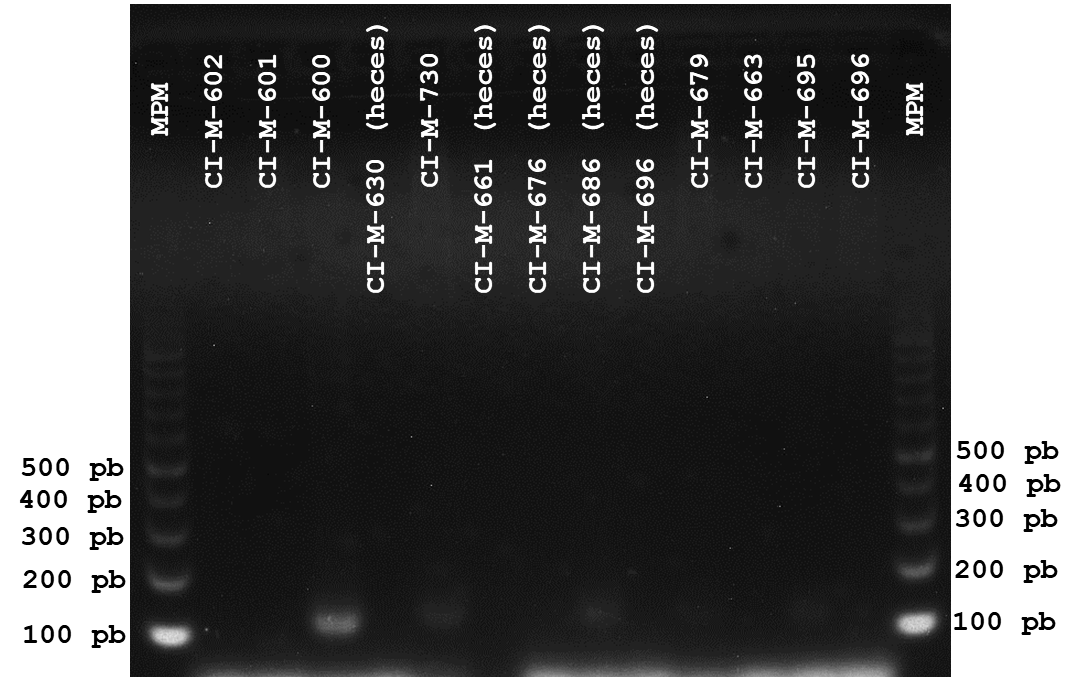


Gel 16. Agarose gel showing PCR products obtained using the Miniexon protocol (Fernandes et al., 2001) for the detection of *Trypanosoma cruzi* in *Triatoma dimidiata*. The expected band (~100 bp) is visible in positive samples. The samples correspond to intestinal content or feces.


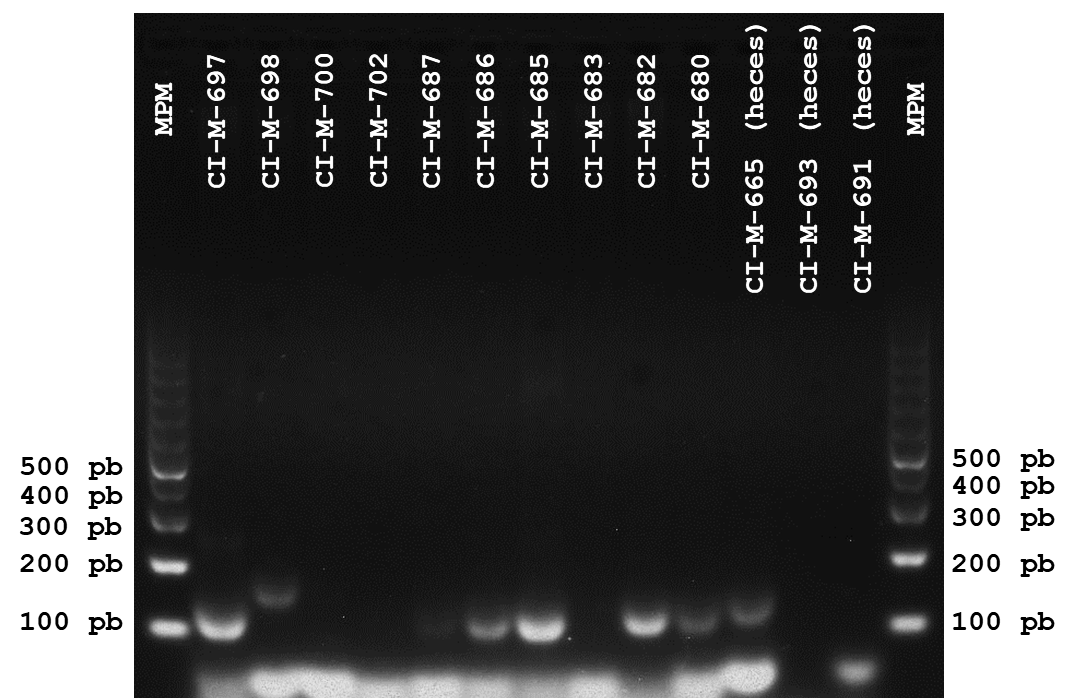


Gel 17. Agarose gel showing PCR products obtained using the Miniexon protocol (Fernandes et al., 2001) for the detection of *Trypanosoma cruzi* in *Triatoma dimidiata*. The expected band (~100 bp) is visible in positive samples. The samples correspond to intestinal content or feces.
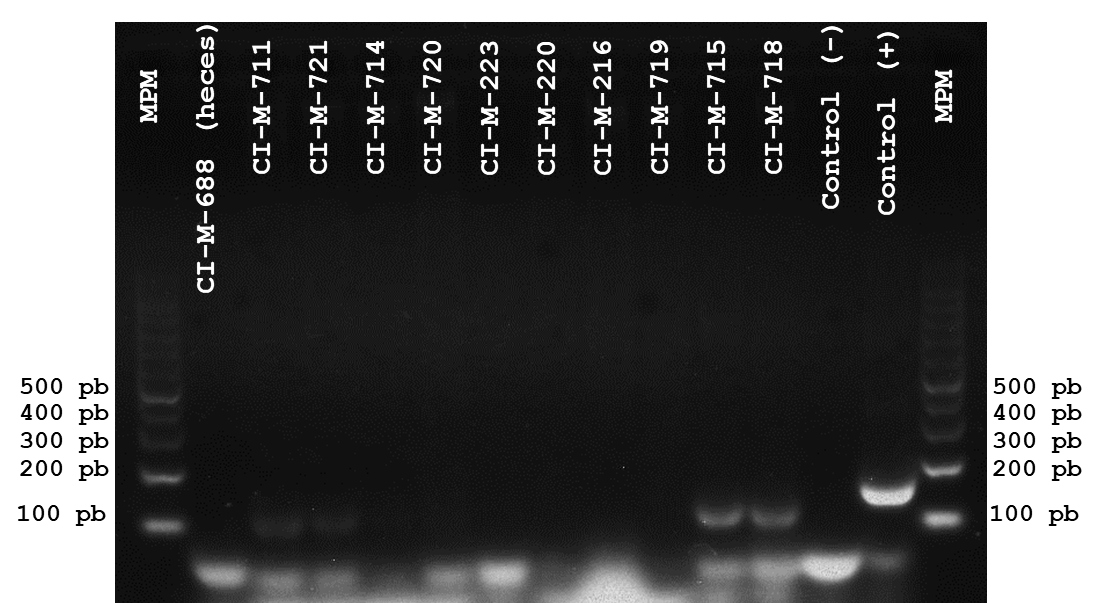


Gel 18. Agarose gel showing PCR products obtained using the Miniexon protocol (Fernandes et al., 2001) for the detection of *Trypanosoma cruzi* in *Triatoma dimidiata*. The expected band (~100 bp) is visible in positive samples. The samples correspond to intestinal content or feces.
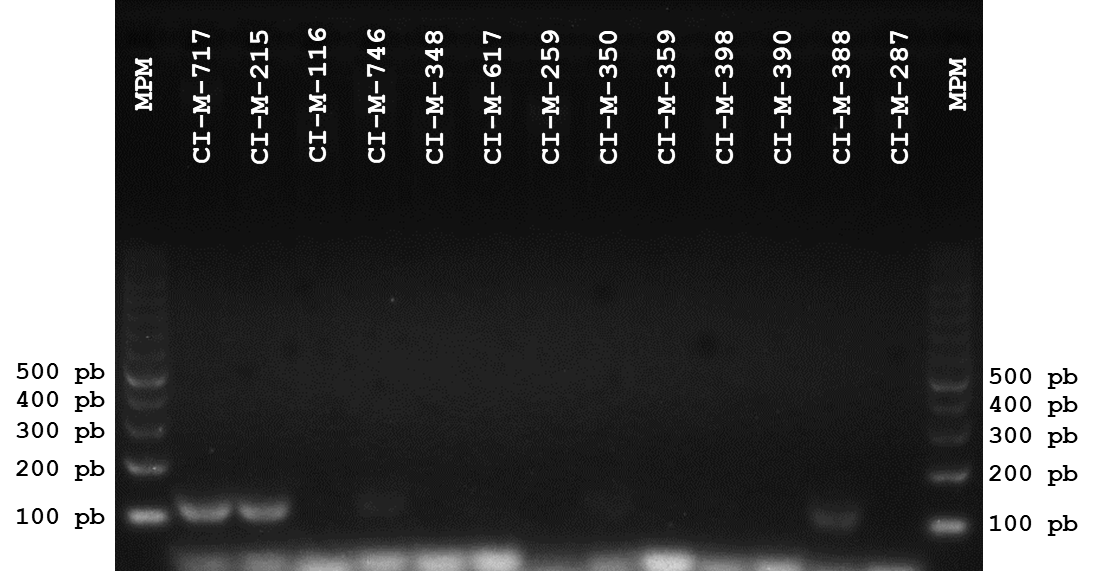


Gel 19. Agarose gel showing PCR products obtained using the Miniexon protocol (Fernandes et al., 2001) for the detection of *Trypanosoma cruzi* in *Triatoma dimidiata*. The expected band (~100 bp) is visible in positive samples. The samples correspond to intestinal content or feces.
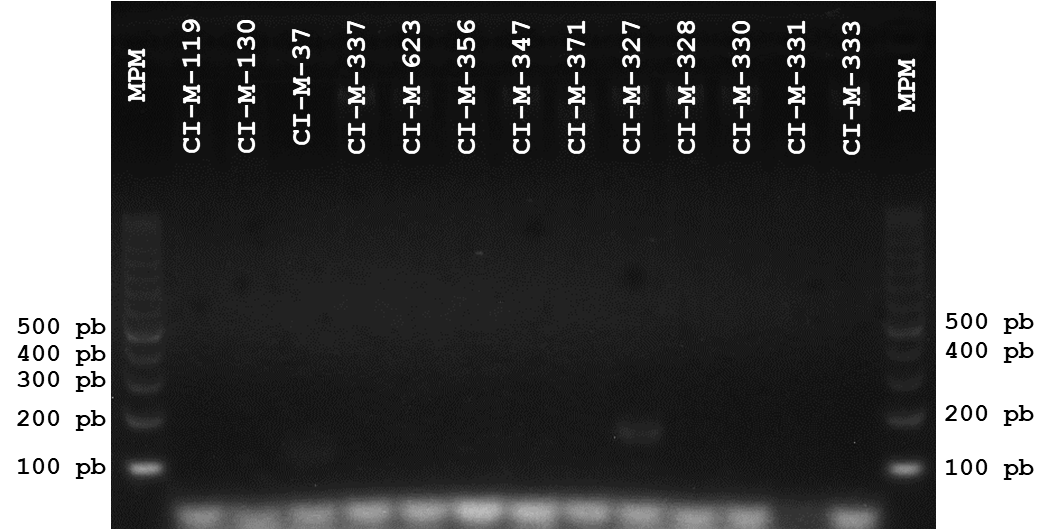


Gel 20. Agarose gel showing PCR products obtained using the Miniexon protocol (Fernandes et al., 2001) for the detection of *Trypanosoma cruzi* in *Triatoma dimidiata*. The expected band (~100 bp) is visible in positive samples. The samples correspond to intestinal content or feces.
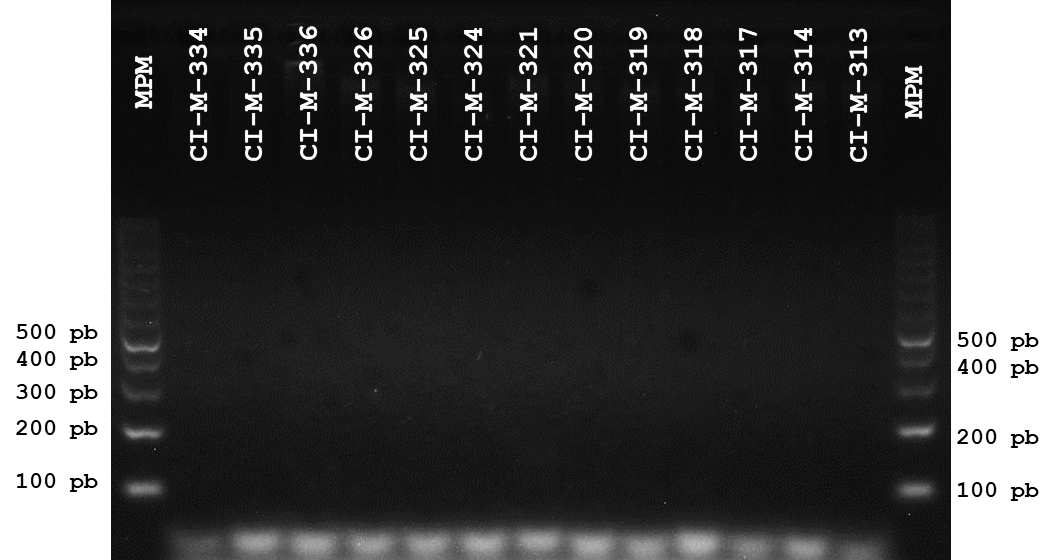


Gel 21. Agarose gel showing PCR products obtained using the Miniexon protocol (Fernandes et al., 2001) for the detection of *Trypanosoma cruzi* in *Triatoma dimidiata*. The expected band (~100 bp) is visible in positive samples. The samples correspond to intestinal content or feces.


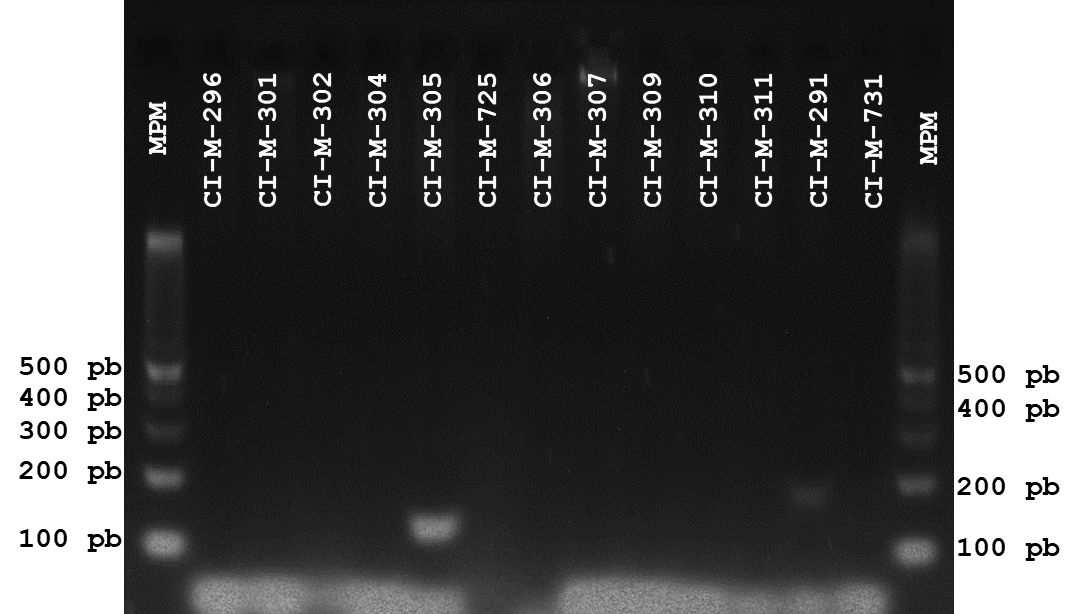


Gel 22. Agarose gel with PCR products obtained using the Miniexon protocol (Fernandes et al., 2001) for the detection of *Trypanosoma cruzi* in *Triatoma dimidiata* samples. The expected band (~100 bp) is visible in positive samples. The samples correspond to intestinal contents or feces.


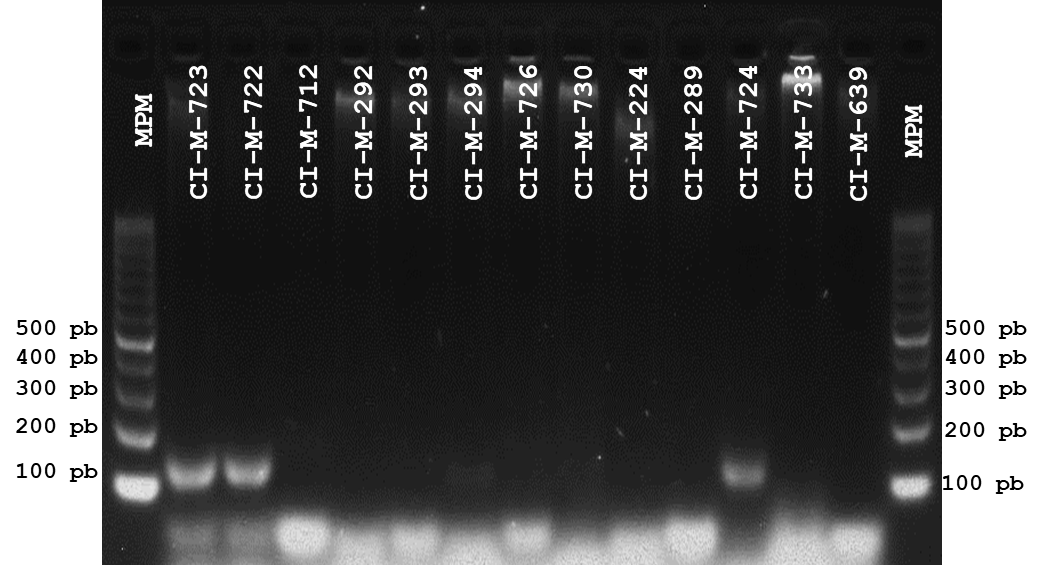


Gel 23. Agarose gel with PCR products obtained using the Miniexon protocol (Fernandes et al., 2001) for the detection of *Trypanosoma cruzi* in *Triatoma dimidiata*. The expected band (~100 bp) is visible in positive samples. The samples correspond to intestinal contents or feces.


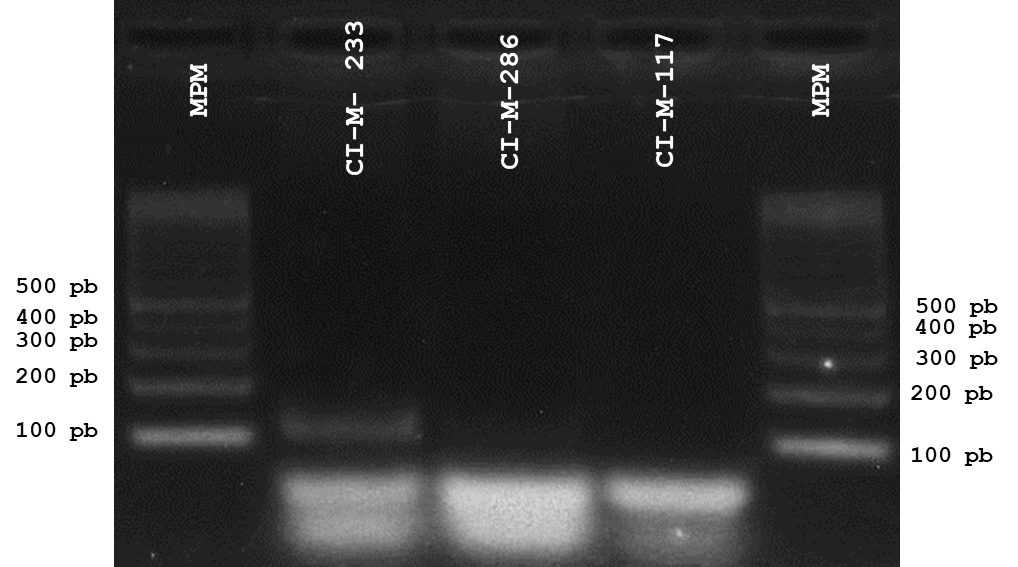


Gel 24. Agarose gel showing PCR products obtained and amplified from the small subunit ribosomal RNA gene using the 18S protocol (Noyes et al., 1996) for the detection of *Trypanosoma cruzi* in mammal samples. The expected band (~500 bp) is visible in positive samples. The table indicates the sample code and the corresponding mammal species.

| Up | |  | Down | |
| --- | --- | --- | --- | --- |
| Code | Specie |  | Code | Specie |
| KK032 | *Artibeus jamaicensis* |  | KK323 | *Pteronotus parnellii* |
| KK137 | *Sturnira parvidens* |  | KK216 | *Desmodus rotundus* |
| KK321 | *Pteronotus parnellii* |  | KK222 | *Artibeus jamaicensis* |
| KK112 | *Pteronotus parnellii* |  | KK302 | *Artibeus jamaicensis* |
| KK363 | *Artibeus jamaicensis* |  | KK328 | *Ototylomys phyllotis* |
| KK108 | *Mormoops megalophylla* |  | KK197 | *Pteronotus davyi* |
| KK038 | *Artibeus jamaicensis* |  | KK405 | *Pteronotus davyi* |
| KK399 | *Pteronotus davyi* |  | KK425 | *Pteronotus parnellii* |
| KK205 | *Artibeus jamaicensis* |  | KK143 | *Pteronotus parnellii* |
| KK224 | *Desmodus rotundus* |  | KK113 | *Pteronotus parnellii* |
| KK019 | *Dermanura phaeotis* |  | KK336 | *Pteronotus parnellii* |
| KK048 | *Artibeus jamaicensis* |  | KK368 | *Artibeus jamaicensis* |
| KK228 | *Desmodus rotundus* |  | KK055 | *Dermanura tolteca* |
| KK374 | *Artibeus jamaicensis* |  | KK412 | *Pteronotus parnellii* |
| KK336 | *Pteronotus parnellii* |  |  |  |
| KK374 | *Artibeus jamaicensis* |  |  |  |
| KK336 | *Pteronotus parnellii* |  |  |  |
| KK317 | *Artibeus jamaicensis* |  |  |  |


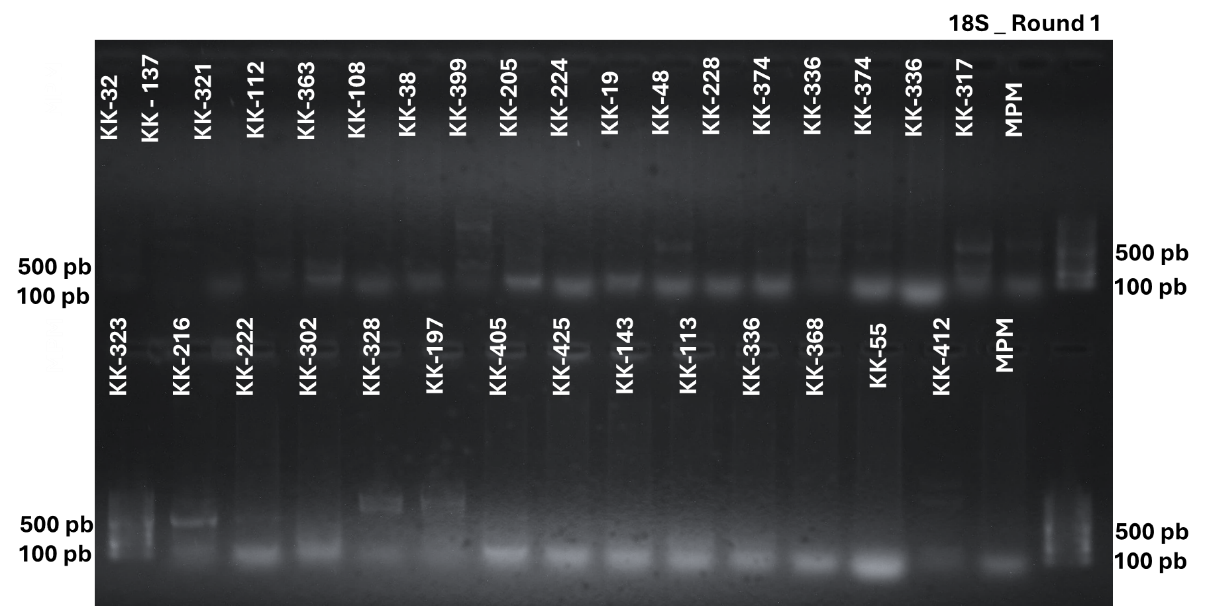


Gel 25. Agarose gel showing PCR products obtained and amplified from the small subunit ribosomal RNA gene using the 18S protocol (Noyes et al., 1996) for the detection of *Trypanosoma cruzi* in mammal samples. The expected band (~500 bp) is visible in positive samples. The table indicates the sample code and the corresponding mammal species.

| Up | |  | Down | |
| --- | --- | --- | --- | --- |
| Code | Specie |  | Code | Specie |
| KK117 | *Pteronotus parnellii* |  | KK318 | *Pteronotus parnellii* |
| KK350 | *Artibeus jamaicensis* |  | KK417 | *Pteronotus parnellii* |
| KK400 | *Pteronotus davyi* |  | KK034 | *Artibeus jamaicensis* |
| KK367 | *Artibeus jamaicensis* |  | KK195 | *Desmodus rotundus* |
| KK311 | *Artibeus jamaicensis* |  |  |  |
| KK310 | *Pteronotus parnellii* |  |  |  |
| KK029 | *Artibeus jamaicensis* |  |  |  |
| KK277 | *Mormoops megalophylla* |  |  |  |
| KK369 | *Artibeus jamaicensis* |  |  |  |
| KK111 | *Pteronotus parnellii* |  |  |  |
| KK396 | *Pteronotus parnellii* |  |  |  |
| KK365 | *Artibeus jamaicensis* |  |  |  |
| KK348 | *Artibeus jamaicensis* |  |  |  |


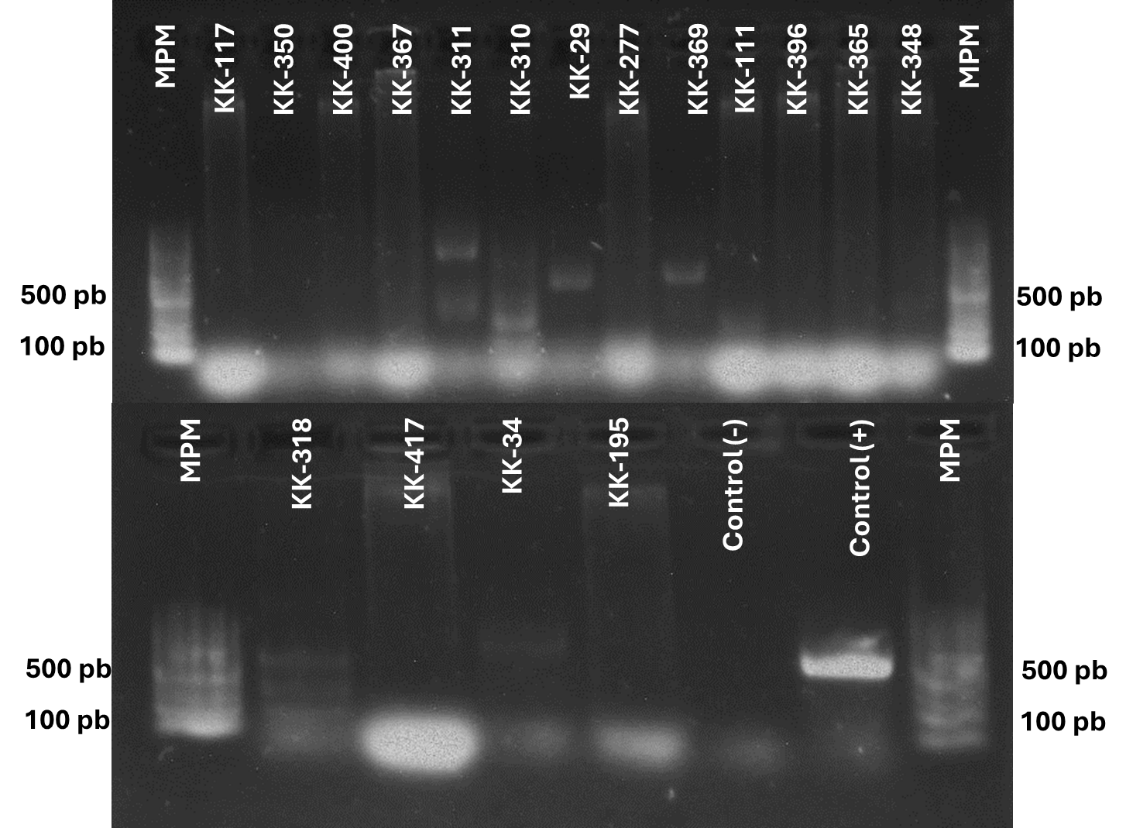


Gel 26. Agarose gel showing PCR products obtained and amplified from the small subunit ribosomal RNA gene using the 18S protocol (Noyes et al., 1996) for the detection of *Trypanosoma cruzi* in mammal samples. The expected band (~500 bp) is visible in positive samples. The table indicates the sample code and the corresponding mammal species. The first five samples correspond to mammals. The remaining samples correspond to intestinal contents or feces of *Triatoma dimidiata*.

| Clave | Especie |
| --- | --- |
| KK410 | *Pteronotus davyi* |
| KK223 | *Desmodus rotundus* |
| KK214 | *Molossus rufus* |
| KK414 | *Pteronotus davyi* |
| KK316 | *Pteronotus parnellii* |


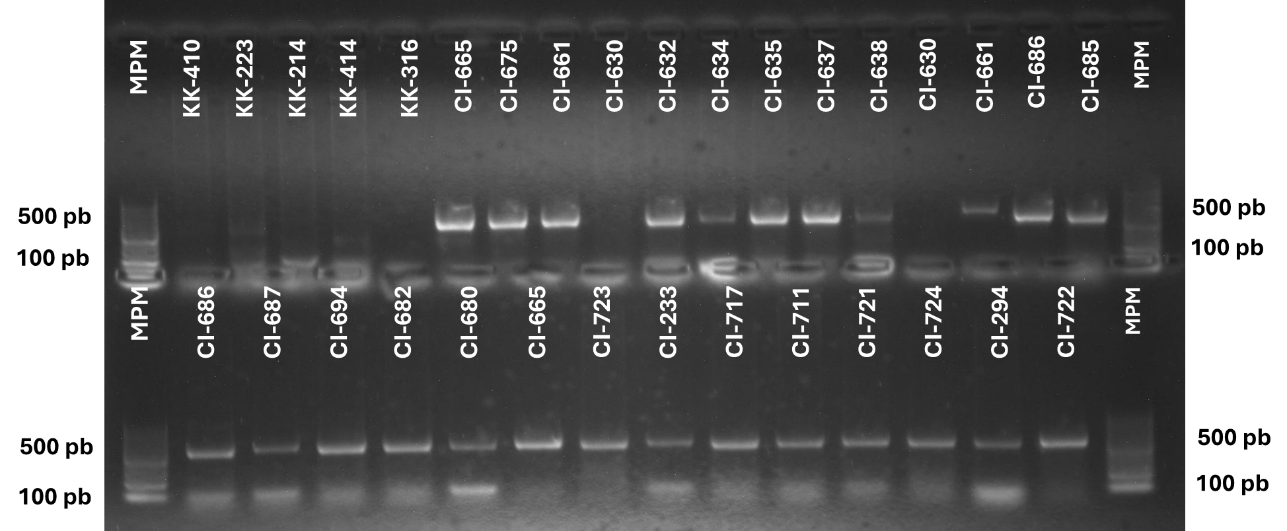


Gel 27. Agarose gel showing PCR products obtained and amplified from the small subunit ribosomal RNA gene using the 18S protocol (Noyes et al., 1996) for the detection of *Trypanosoma cruzi* in mammal samples. The expected band (~500 bp) is visible in positive samples. The table indicates the sample code and the corresponding mammal species. Samples labeled with “KK” correspond to mammals. The remaining samples correspond to intestinal contents of *Triatoma dimidiata*, except for those indicated, which were obtained from feces.

| Clave | Especie |
| --- | --- |
| KK159 | *Heteromys gaumeri* |
| KK185 | *Heteromys gaumeri* |
| KK160 | *Heteromys gaumeri* |
| KK189 | *Heteromys gaumeri* |
| KK191 | *Heteromys gaumeri* |
| KK247 | *Heteromys gaumeri* |
| KK284 | *Heteromys gaumeri* |


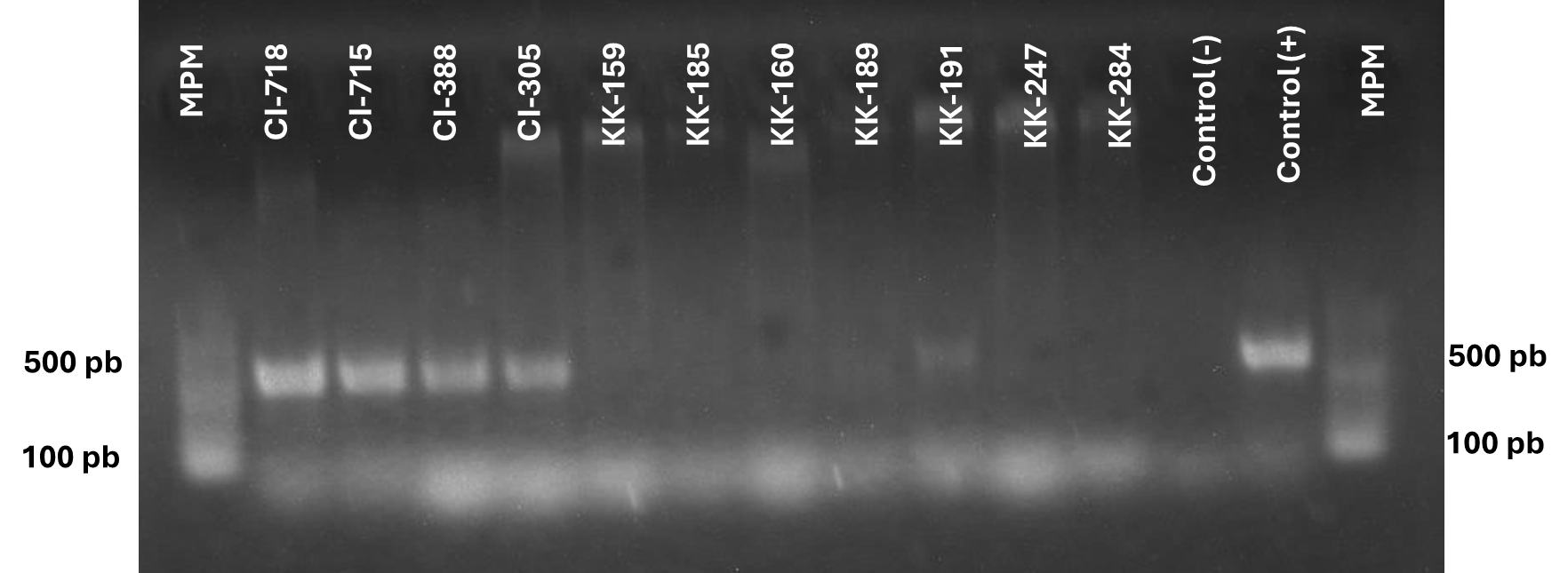


Gel 28. Agarose gel showing PCR products obtained and amplified from the small subunit ribosomal RNA gene using the 18S protocol (Noyes et al., 1996) for the detection of *Trypanosoma cruzi* in mammal samples. The expected band (~500 bp) is visible in positive samples. The table indicates the sample code and the corresponding mammal species.

| Code | Species |
| --- | --- |
| KK032 | *Artibeus jamaicensis* |
| KK137 | *Sturnira parvidens* |
| KK038 | *Artibeus jamaicensis* |
| KK205 | *Artibeus jamaicensis* |
| KK204 | *Artibeus jamaicensis* |
| KK019 | *Dermanura phaeotis* |
| KK048 | *Artibeus jamaicensis* |
| KK228 | *Desmodus rotundus* |
| KK336 | *Pteronotus parnellii* |
| KK317 | *Artibeus jamaicensis* |
| KK323 | *Pteronotus parnellii* |


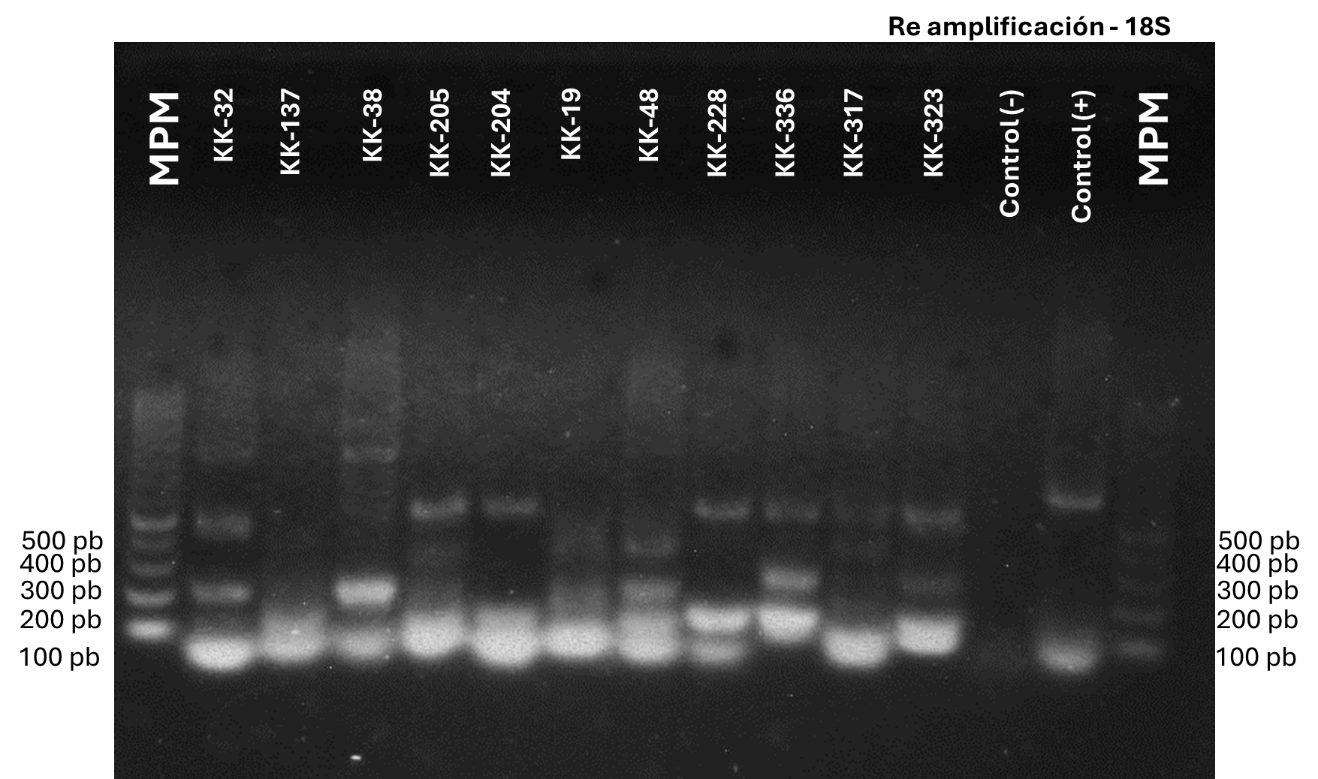


Gel 29. Agarose gel showing PCR products obtained and amplified from the small subunit ribosomal RNA gene using the 18S protocol (Noyes et al., 1996) for the detection of *Trypanosoma cruzi* in mammal samples. The expected band (~500 bp) is visible in positive samples. The table indicates the sample code and the corresponding mammal species. Samples labeled with "KK" correspond to mammals. The remaining samples correspond to intestinal contents or feces from *Triatoma dimidiata*.

| Code | Species |
| --- | --- |
| KK216 | *Desmodus rotundus* |
| KK302 | *Artibeus jamaicensis* |
| KK328 | *Ototylomys phyllotis* |
| KK055 | *Dermanura tolteca* |
| KK311 | *Artibeus jamaicensis* |
| KK029 | *Artibeus jamaicensis* |
| KK369 | *Artibeus jamaicensis* |
| KK318 | *Pteronotus parnellii* |
| KK034 | *Artibeus jamaicensis* |
| KK360 | *Artibeus jamaicensis* |
| KK371 | *Dermanura phaeotis* |
| KK224 | *Desmodus rotundus* |
| KK276 | *Pteronotus parnellii* |
| KK383 | *Heteromys gaumeri* |
| KK253 | *Heteromys gaumeri* |
| KK191 | *Heteromys gaumeri* |


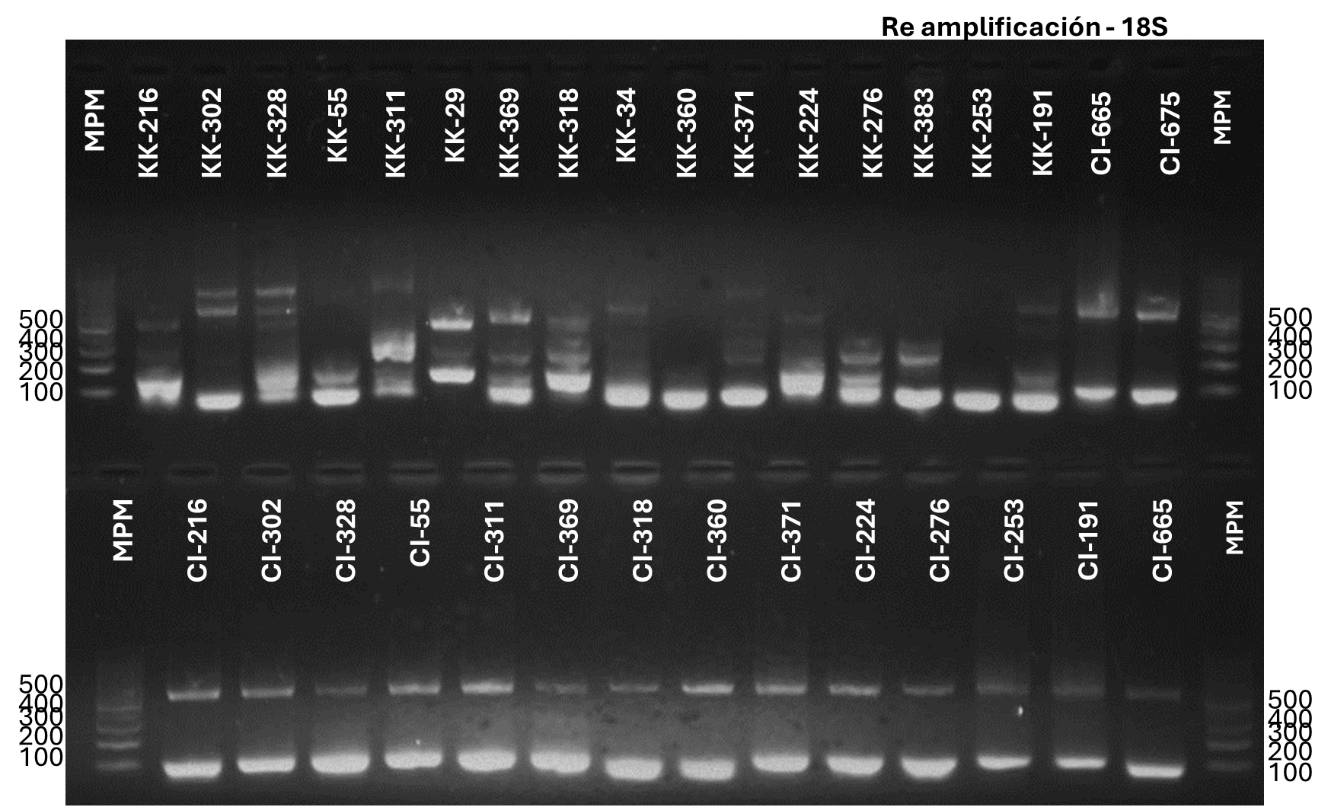


Gel 30. Agarose gel showing PCR products obtained and amplified from the small subunit ribosomal RNA gene using the 18S protocol (Noyes et al., 1996) for the detection of *Trypanosoma cruzi* in *Triatoma dimidiata*. The expected band (~500 bp) is visible in positive samples.
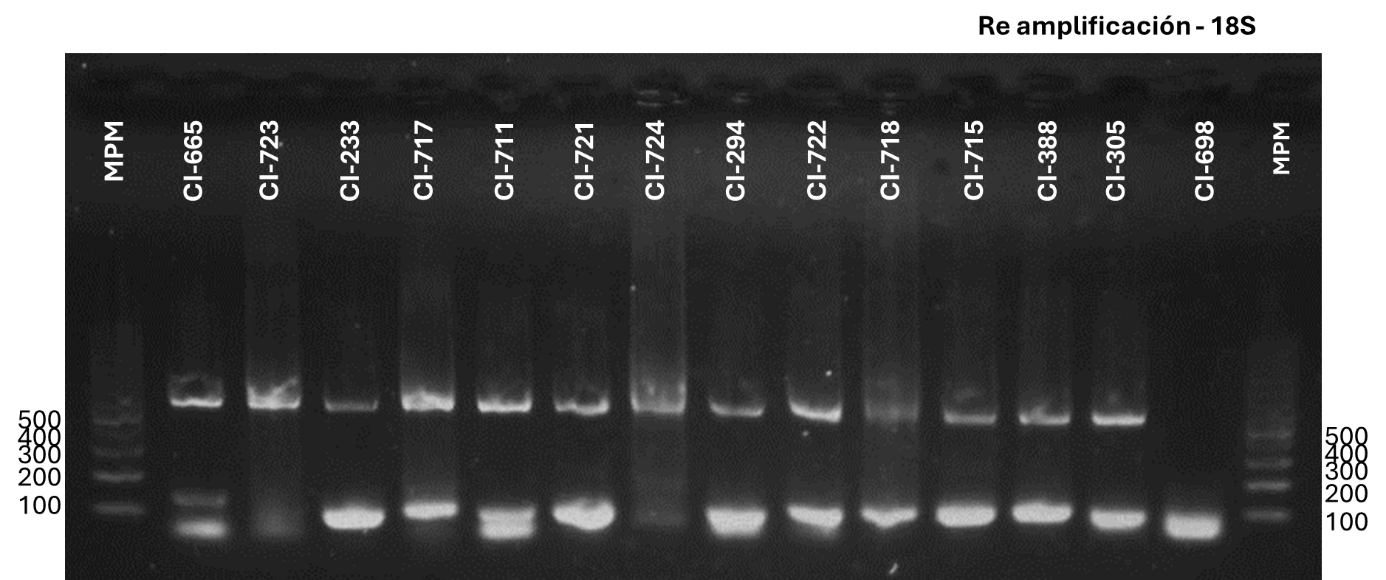


Gel 31. Agarose gel with PCR products amplified using the Miniexon protocol (Fernandes et al., 2001) for the detection of *Trypanosoma cruzi* in *Triatoma dimidiata* samples. The gel shows the amplified samples selected for further analysis.


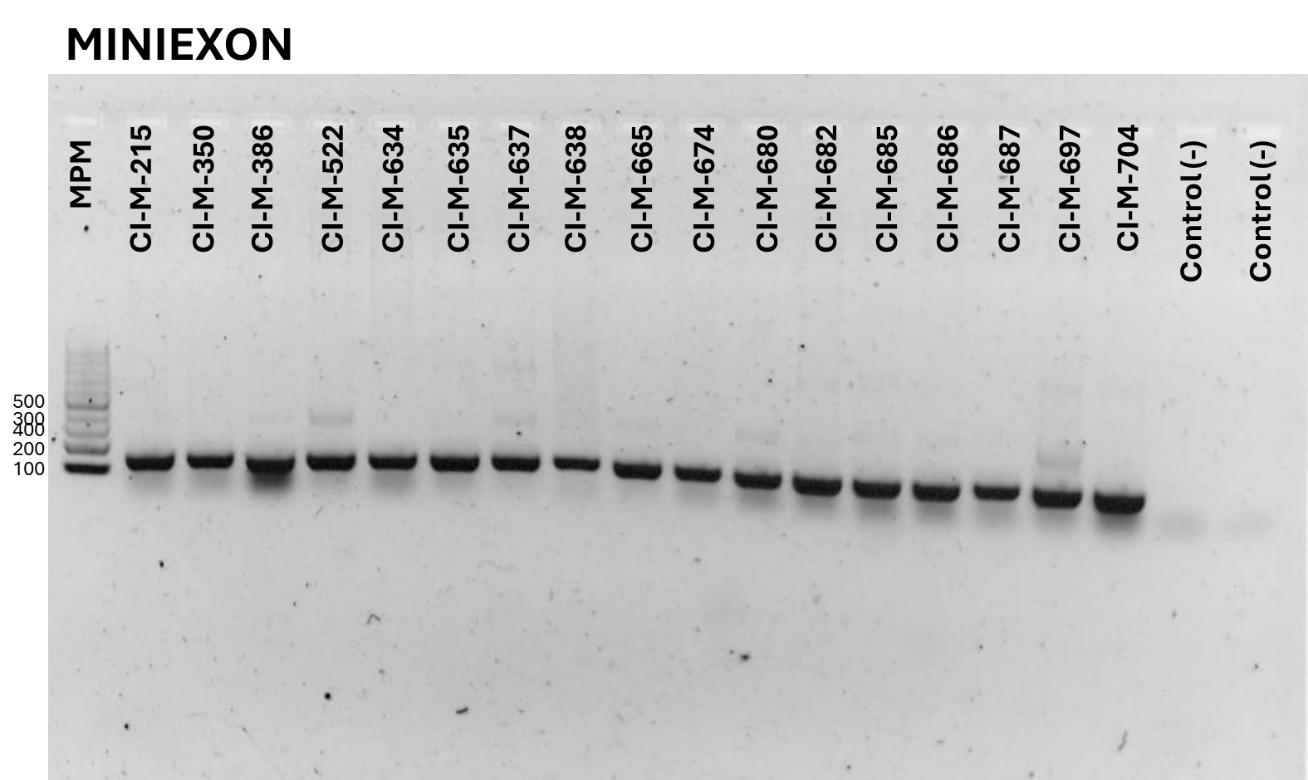


Gel 32. Agarose gel with PCR products amplified using the Miniexon protocol (Fernandes et al., 2001) for the detection of *Trypanosoma cruzi* in *Triatoma dimidiata* and mammal samples. The gel shows the amplified samples selected for further analysis.

| Code | Species |
| --- | --- |
| KK399 | *Pteronotus davyi* |
| KK405 | *Pteronotus davyi Pteronotus davyi* |
| KK410 | *Pteronotus davyi* |
| KK316 | *Pteronotus parnellii* |
| KK048 | *Artibeus jamaicensis* |
| KK340 | *Heteromys gaumeri* |


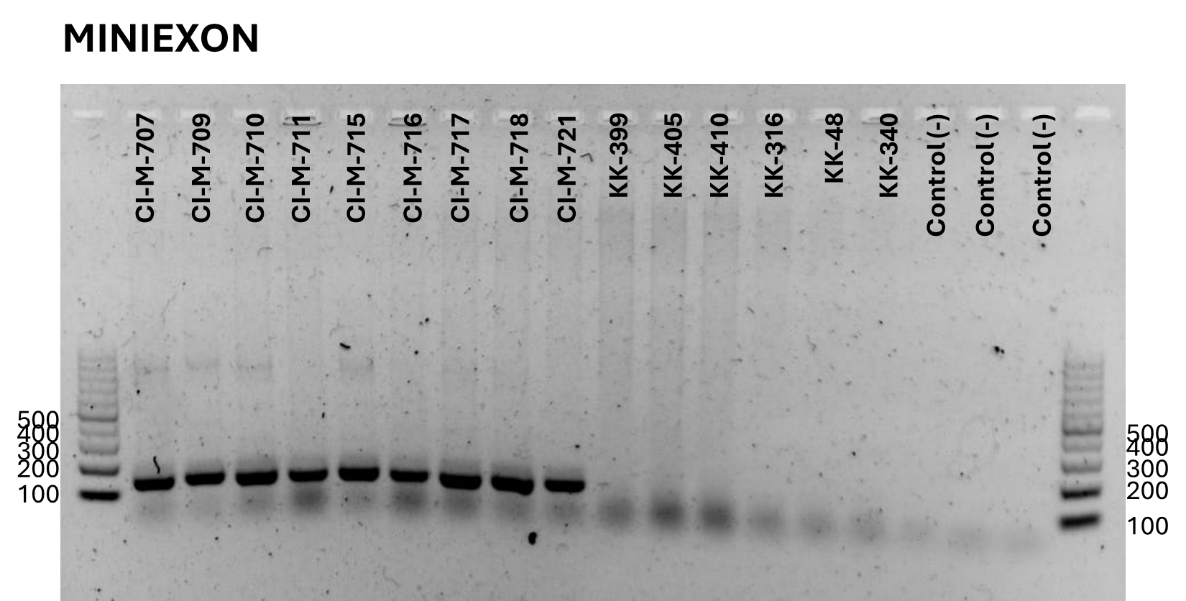


Gel 33. PCR products amplified from the small subunit ribosomal RNA gene for the detection of Trypanosoma spp. in mammals

| Code | Species |
| --- | --- |
| KK309 | *Pteronotus parnellii* |
| KK349 | *Pteronotus parnellii* |
| KK025 | *Artibeus jamaicensis* |
| KK046 | *Artibeus jamaicensis* |
| KK126 | *Artibeus jamaicensis* |
| KK241 | *Artibeus jamaicensis* |
| KK271 | *Artibeus jamaicensis* |
| KK274 | *Artibeus jamaicensis* |
| KK279 | *Artibeus jamaicensis* |
| KK032 | *Artibeus jamaicensis* |
| KK205 | *Artibeus jamaicensis* |
| KK228 | *Desmodus rotundus* |
| KK336 | *Pteronotus parnellii* |
| KK323 | *Pteronotus parnellii* |
| KK311 | *Artibeus jamaicensis* |


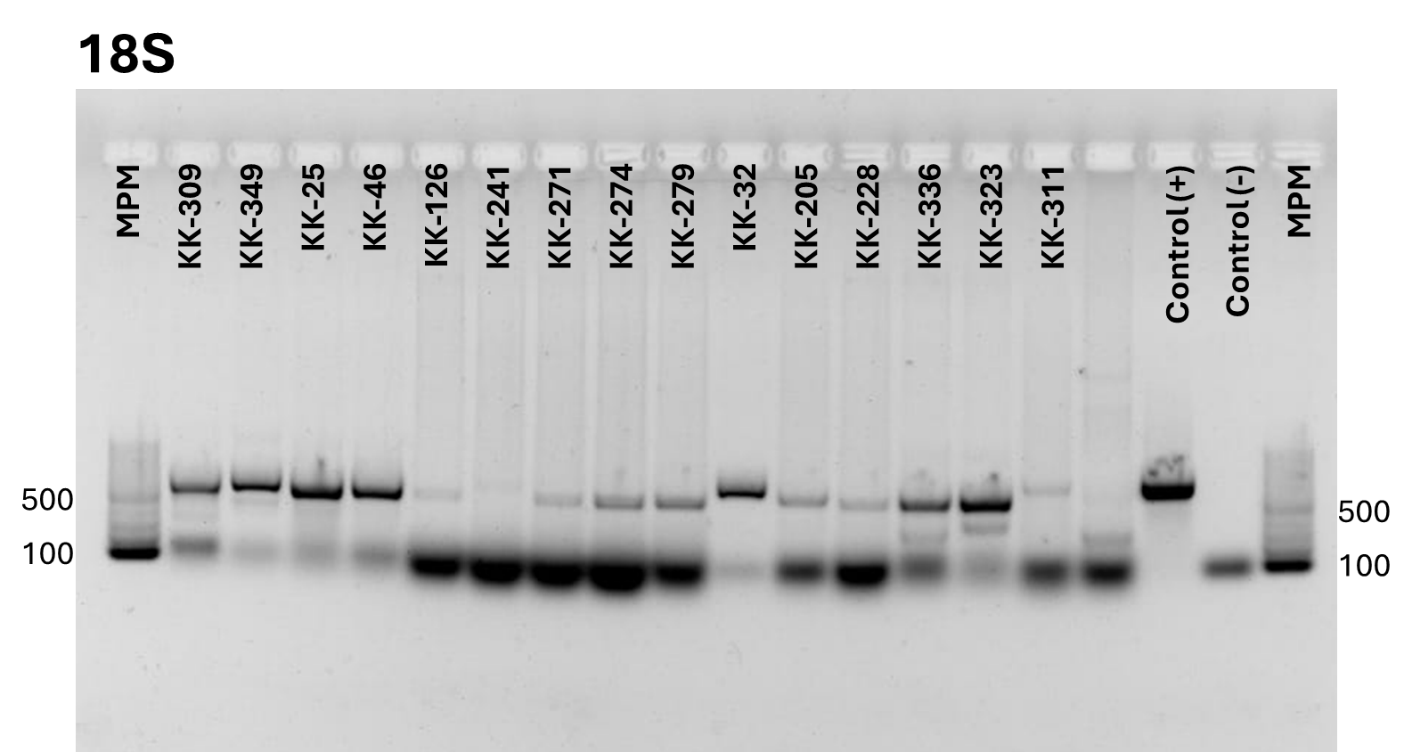

Supplement: Supplementary file 2 — Supplementary Material 2. (DOCX 15.2 MB) [file 436_2026_8670_MOESM2_ESM.docx]
